# Alcohol-sourced acetate impairs T cell function by promoting cortactin acetylation

**DOI:** 10.1016/j.isci.2023.107230

**Published:** 2023-06-28

**Authors:** Vugar Azizov, Michel Hübner, Michael Frech, Jörg Hofmann, Marketa Kubankova, Dennis Lapuente, Matthias Tenbusch, Jochen Guck, Georg Schett, Mario M. Zaiss

**Affiliations:** 1 Department of Internal Medicine 3, Rheumatology and Immunology, Friedrich-Alexander-University Erlangen-Nürnberg and Universitätsklinikum Erlangen, Erlangen, Germany; 2 Deutsches Zentrum Immuntherapie (DZI), Friedrich-Alexander-University Erlangen-Nürnberg and Universitätsklinikum Erlangen, Erlangen, Germany; 3 Division of Biochemistry, Department of Biology, Friedrich-Alexander-University Erlangen-Nürnberg, Erlangen, Germany; 4 Max Planck Institute for the Science of Light & Max Planck Zentrum für Physik und Medizin, Erlangen, Germany; 5 Institute of Clinical and Molecular Virology, Friedrich-Alexander-University Erlangen-Nürnberg and Universitätsklinikum Erlangen, Erlangen, Germany

**Keywords:** Immunology, Molecular biology

## Abstract

Alcohol is among the most widely consumed dietary substances. Excessive alcohol consumption damages the liver, heart, and brain. Alcohol also has strong immunoregulatory properties. Here, we report how alcohol impairs T cell function via acetylation of cortactin, a protein that binds filamentous actin and facilitates branching. Upon alcohol consumption, acetate, the metabolite of alcohol, accumulates in lymphoid organs. T cells exposed to acetate, exhibit increased acetylation of cortactin. Acetylation of cortactin inhibits filamentous actin binding and hence reduces T cell migration, immune synapse formation and activation. While mutated, acetylation-resistant cortactin rescues the acetate-induced inhibition of T cell migration, primary mouse cortactin knockout T cells exhibited impaired migration. Acetate-induced cytoskeletal changes effectively inhibited activation, proliferation, and immune synapse formation in T cells *in vitro* and *in vivo* in an influenza infection model in mice. Together these findings reveal cortactin as a possible target for mitigation of T cell driven autoimmune diseases.

## Highlights

• Alcohol-sourced acetate contributes to protein acetylation in T cells
• T cell cortactin is acetylated when exposed to alcohol-sourced acetate
• T cell function, migration, and motility is compromised due to cortactin acetylation
• T cell migration and motility is rescued by mutating cortactin acetylation sites

## Graphical abstract

**Figure:**
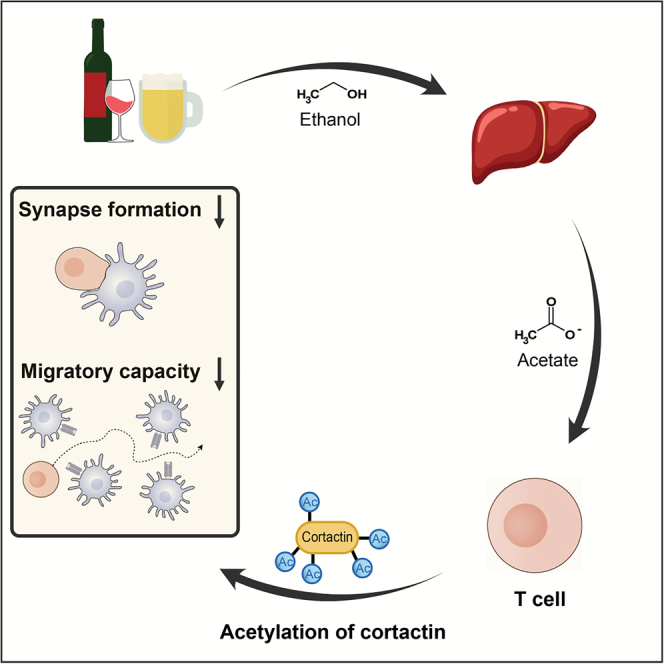

## Introduction

For appropriate immune response to occur, immune cells, such as T cells, must migrate within and outside of lymphoid organs.^1^^,^^2^^,^^3^^,^^4^ The cytoskeleton plays a vital role during T cell migration, scanning for antigens, and cell activation.^5^^,^^6^^,^^7^ To migrate, the cytoskeletal structures must remain highly dynamic.^3^ Failure in cytoskeletal machinery leads to immunodeficiencies due to defects in immune synapse formation, chemotaxis, and migration.^8^ Modulation of the components of cytoskeleton via post-translational modifications such as acetylation provides further fine-tuning to fit cell’s demands.^9^ Histone deacetylases (HDAC) reverse acetylation of variety of proteins and have been shown to impact immunomodulatory effects of T cells.^10^ HDAC6 is one of the deacetylases acting upon cytoskeletal proteins that are required for proper immune synapse formation and migration.^11^^,^^12^^,^^13^^,^^14^^,^^15^^,^^16^ For example, pharmacological inhibition of HDAC6 in a preclinical model of systemic lupus erythematosus (SLE) reduced infiltrating T follicular helper (T_FH_) cells into germinal centers (GCs) with significant consequences on autoantibody titers.^13^^,^^14^ Similarly, we have previously shown that increased exposure to alcohol and alcohol’s main metabolite, acetate, affect T_FH_ cell responses in the collagen induced arthritis (CIA)—a mouse model of inflammatory arthritis.^17^ Mice exposed to either alcohol-sourced or directly supplemented acetate exhibited reduced T_FH_ cell infiltration of B cell follicles and destabilized T_FH_-B cell conjugate formation both *in vivo* and *in vitro.*^17^ Consequently, we observed acetate’s suppression to be specific to T cell dependent humoral responses both in autoimmune and vaccination mouse models.^17^ Inhibition of HDAC6 in the context of inflammatory arthritis has been shown to reduce disease severity to levels comparable to dexamethasone treatment.^12^ Here, we hypothesized that acetate’s inhibitory effect could be due to increased acetylation of the cytoskeletal proteins ultimately affecting T cell migration and function. Our hypothesis could explain alcohol’s double-edged sword effect in regard to benefits in autoimmunity and damages to health in general.^18^^,^^19^ Alcohol consumption has correlated with decreased severity in rheumatoid arthritis (RA), type 1 diabetes, SLE, and multiple sclerosis in humans as well as in disease mouse models.^19^ Upon consumption, alcohol is first rapidly metabolized to toxic intermediary metabolite acetaldehyde, and then to acetate, contributing to increased serum concentrations.^20^ It is important to note the detrimental health effects and induced molecular changes by acetaldehyde.^19^^,^^21^ In our previous study where we made use of mouse model of alcohol consumption with RA, we reported that mice consuming 10% alcohol (v/v) have average blood alcohol concentration of about 0.03 g per milliliter (0.65 mM).^17^^,^^22^^,^^23^ We also demonstrated an approximate 2-fold increase in blood acetate concentration from 0.04 mM to 0.08 mM upon chronic alcohol consumption.^17^ In contrast to mice, humans demonstrated much higher blood alcohol and consequent blood acetate concentrations reaching approximately 2 mM concentrations upon alcohol consumption.^24^^,^^25^ Our previous measurements in steady state mice, showed 0.04 mM acetate concentration in serum, while literature reported data in humans, shows about 0.5 mM.^17^^,^^24^ While there is no data on acetate accumulation in human tissues upon alcohol consumption, akin to our mouse data, there is about 2x fold increase in serum acetate levels of alcoholics.^17^^,^^24^ Once in the blood, acetate permeates cells where it is converted to acetyl-Co-enzyme A (acetyl-CoA) and used as a donor for protein acetylation.^26^^,^^27^ Indeed, increased levels of intracellular acetyl-CoA were linked with significantly higher protein acetylation.^28^ In 2019, Mews and colleagues showed that alcohol consumption can quickly lead to histone acetylation in the brain.^29^ Considering worldwide use of alcohol, its contribution to rise in blood acetate concentrations, and acetate’s potential to increase acetylation of intracellular proteins, we set out to investigate whether there is a link between acetate exposure and mitigation of cytoskeletal dynamics in T cells.

Our findings indicate that cortactin, previously not known to play a key role in T cell function, is expressed in T cells. We show that upon alcohol consumption, alcohol metabolism leads to increased acetate levels and accumulation in lymphoid organs. Consequently, T cells that are exposed to increased acetate concentrations, demonstrate increased acetylation of cortactin, leading to decreased F-actin binding. In turn we observed deficiencies in lamellipodia formation, *in vitro* and *in vivo* migration, and T cell activation. We also demonstrate reduced T cell function in alcohol consuming influenza infected mice.

## Results

### Upon alcohol consumption acetate accumulates in lymphoid organs

**Figure 1:**
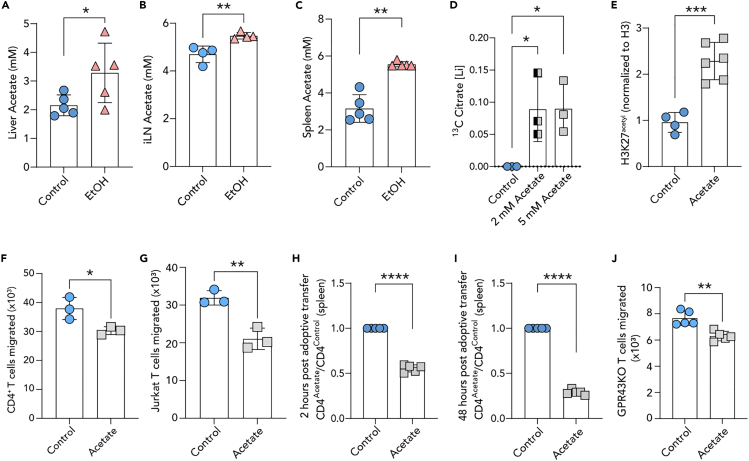
Acetate accumulates in lymphoid organs and impairs T cells migration capacity (A–C) GC/MS quantification of acetate in (A) livers, (B) inguinal lymph nodes (iLN), and (C) spleens of alcohol-fed mice compared with water-fed control mice, expressed in mM concentration (individual data point represents one mouse). (D) Mouse CD4^+^ T cells treated *in vitro* with 5 mM ^13^C labeled acetate exhibit increased ^13^C labeled citrate, quantified by GC-MS. (E) Quantification of western blot analysis of the level of acetylated histone 3 at lysine 27 of mouse CD4^+^ T cells treated *in vitro* with 5 mM acetate (also see Figure S1A). (F and G) Numbers of migrated mouse naive CD4^+^ T cells and (G) Jurkat T cells *in vitro trans*-well assay, counted by flow cytometer. (H) The ratio of acetate to control treated mouse CD4^+^ T cells found in the spleens of recipient mice in an adoptive transfer experiment at 2 h and (I) 48 h post transfer (each point represents one mouse, n = 5) (also see Figure S1B). (J) Numbers of migrated mouse GPR43KO CD4^+^ T cells treated with 5 mM acetate in an *in vitro trans*-well assay, counted by flow cytometry. Representative data shown are either from one of two independent experiments (A–C, E, H–J) or combined three independent experiments (D), or one of three independent experiments (F, G), and expressed as mean ± SD. Statistical difference was determined by Student’s two-tailed t-test. ∗p < 0.05; ∗∗p < 0.01; ∗∗∗p < 0.001; ∗∗∗∗p < 0.0001.

Building upon our published results, we ventured to study whether the decreased infiltration of GCs by T and B cells along with reduced T_FH_-B cell contacts was due to cytoskeletal deficiencies following alcohol-sourced acetate exposure.^17^ First, we set to quantify the exposure of T cells to acetate upon alcohol consumption by measuring acetate levels in alcohol-impacted and immune relevant lymphoid tissues such as the liver, inguinal lymph node (iLN), and spleen of alcohol-fed mice. We found that acetate indeed accumulates in these organs, especially in lymphoid organs reaching concentrations surpassing 5 mM (Figures 1A–1C). Next, we quantified whether exposure to 5 mM acetate concentration results in incorporation to cellular metabolism. Previously it was shown that increased *in vivo* ethanol concentration can lead to an increase in intracellular acetate and citrate.^29^ In an *in vitro* treatment of mouse naive CD4^+^ CD25^−^ CD44^low^ CD62L^high^ T cells, hereafter naive CD4^+^ T cells, with ^13^C labeled acetate, we were able to confirm an increase in ^13^C containing citrate starting at exposure to 2 mM acetate concentrations (Figure 1D). In a subsequent quantification of histone 3 (H3) acetylation levels in the same CD4^+^ T cells, we observed increased H3 acetylated at lysine 27 (Figures 1E and S1A). Together, these findings confirm increased exposure of T cells to acetate, leading to increased protein acetylation within cells.

### Exposure to acetate reduces T cell migration capacity

We next performed a *trans*-well migration assay of either mouse CD4^+^ or human Jurkat T cells in the presence and absence of acetate. Here, we found a 20%–30% decrease in *trans*-well migration of mouse and human derived CD4^+^ T cells (Figures 1F and 1G). To study the potential impact of decreased T cell migration *in vivo*, primary mouse CD4^+^ T cells were pre-treated for 4 h with 5 mM acetate *in vitro*, loaded with CellTrace dye and combined at 1:1 ratio of control to acetate treated CD4^+^ T cells, to a total of 6×10^7^ cells. We, then, adoptively transferred 6×10^7^ cells to recipient C57BL/6 wild type naive mice not supplemented with alcohol. Two hours post adoptive transfer, we observed about 50% decrease in migration of acetate treated CD4^+^ T cells to spleens of recipient mice compared to control CD4^+^ T cells (Figures 1H and S1B). The same observation was true at 48 h post adoptive transfer (Figures 1I and S1B). Next, we asked whether acetate exerts its effects on T cells via G-protein coupled receptor 43 (GPR43), a membrane receptor bound and activated by short-chain fatty acids (SCFA).^30^ We found GPR43 knockout (GPR43KO) CD4^+^ T cells also migrate less in an *in vitro trans*-well assay in the presence of acetate (Figure 1J). These data indicate that the exposure to acetate reduces T cell migration capacity *in vitro* and *in vivo*.

### T cells exposed to acetate exhibit reduced total filamentous actin

**Figure 2:**
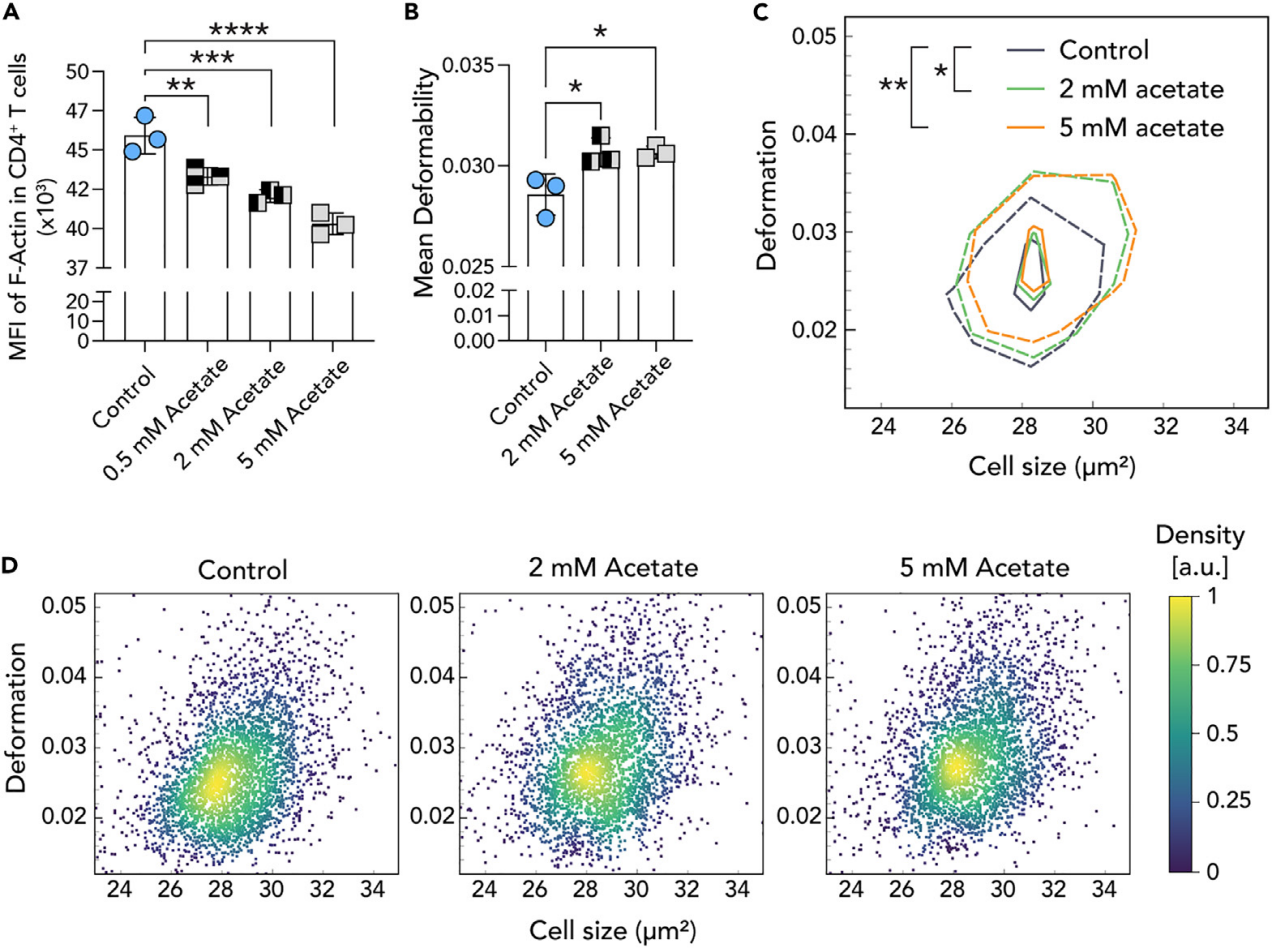
Acetate exposure reduces F-actin and affects T cell deformability (A) Flow cytometry analysis of mouse CD4^+^ T cells shows a dose dependent decrease in phalloidin-Alexa Fluor-488 median fluorescence intensity (MFI) (also see Figure S2). (B) A real-time deformability cytometry (RT-DC) of mouse CD4^+^ T cells shows an increase in deformability upon 2 mM acetate and 5 mM acetate treatment (n = 3, CD4^+^ T cells isolated from separate mice). (C and D) Overlay of the mean values for control, 2 mM and 5 mM acetate treated CD4^+^ T cell deformation and cell size represented in (D) scatterplots of RT-DC analysis of control and acetate treated (2 mM and 5 mM) naive CD4^+^ T cell deformation shown in Figure 2C (n = 2000 cells). Representative data shown from either one of three independent experiments (A) or one of two independent experiments (B, C, D) and expressed as mean ± SD. Statistical difference was determined by one-way ANOVA (A, B). Statistical analyses of panel C were carried out using a one-dimensional linear mixed model that incorporates fixed effect parameters and random effects to analyze differences between cell subsets and replicate variances, respectively. p-values were determined by a likelihood ratio test, comparing the full model with a model lacking the fixed effect term. ∗p < 0.05; ∗∗p < 0.01; ∗∗∗p < 0.001; ∗∗∗∗p < 0.0001.

T cells require a dynamic cytoskeleton: rapid modifications of filamentous actin (F-actin), for migration, immune synapse formation and activation.^3^ Upon quantification of total F-actin levels by Alexa Fluor 488-conjugated phalloidin staining and flow cytometry analysis in mouse naive CD4^+^ T cells exposed to 5 mM acetate, we found a dose dependent decrease of F-actin median fluorescence intensity (MFI) (Figures 2A and S2). Most of the F-actin is concentrated to the edges of the cell, also referred to as cortical actin or cortical F-actin, where it serves to help cells maintain and modify shape.^31^ Hence, F-actin deficiency also manifests itself by increased cell deformability measured by real-time deformability cytometry (RT-DC).^32^ We performed RT-DC and found an increase in T cell deformability upon acetate exposure (Figure 2B). Plotting T cell deformability against cell size sustained the decrease in deformability upon acetate exposure (Figures 2C and 2D). Together these data reveal a reduction of total F-actin in acetate-exposed T cells.

### Exposure to acetate increases acetylation of cortactin in T cells

**Figure 3:**
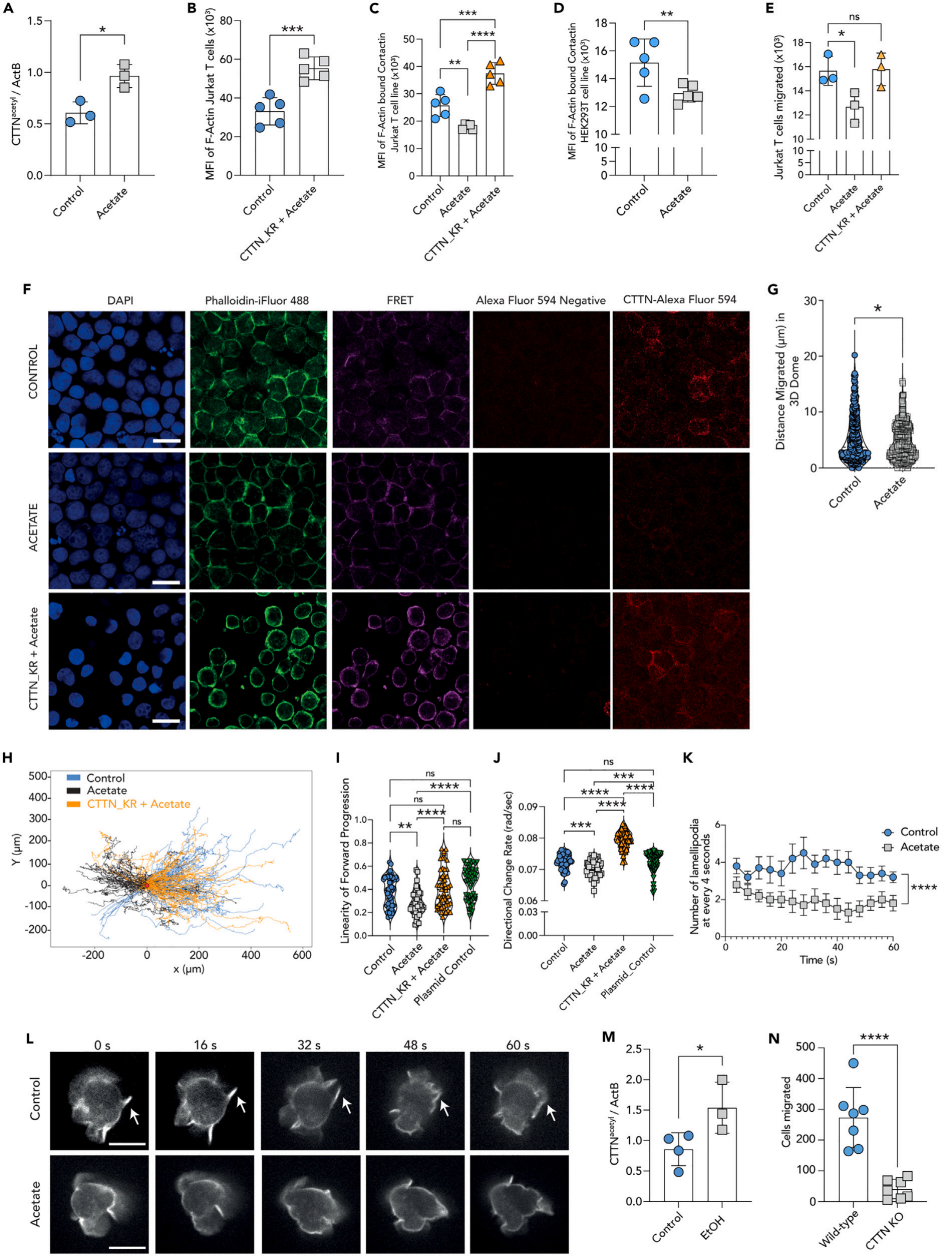
Acetylation-resistant cortactin rescues acetate-induced migration deficiency (A) Quantification of western blot analysis of the level of acetylated cortactin in Jurkat T cells treated with 5 mM acetate, for blot images see Figure S3B. (B) MFI of F-actin in acetate treated Jurkat T cells overexpressing CTTN_KR in comparison to control Jurkat T cells. (C) MFI of Förster resonance energy transfer (FRET) from F-actin bound cortactin in Jurkat T cells quantified by confocal scanning laser microscopy in control and acetate treatment, as well as in acetate treated Jurkat T cells expressing acetylation-resistant cortactin (CTTN_KR), representative images included in panel F. (D) MFI of Förster resonance energy transfer (FRET) from F-actin bound cortactin in control and acetate treated HEK293T cells quantified by confocal laser scanning microscopy. (E) Numbers of migrated Jurkat T cells *in vitro trans*-well assay of control, acetate treated, as well as acetate treated Jurkat T cells expressing acetylation-resistant cortactin (CTTN_KR). (F) Representative images for FRET analysis of cortactin (Alexa Fluor 594), F-actin (Phalloidin-iFluor 488) in Jurkat T cells treated either with vehicle (control) or acetate, and acetate treated Jurkat T cells expressing CTTN_KR. Scale bar = 15 μm. (G) Analysis of Jurkat T cell migration within 3D matrix made of Matrigel by Leica Thunder 3D microscopy, see Video S1 (control), and Video S2 (acetate) (n > 2000 cells analyzed per condition). (H) Individual tracks analyzed in panels G and H. (I and J) Linearity of forward progression and (J) directional change rate of Jurkat T cells treated with 5 mM acetate, medium control, plasmid electroporation control and Jurkat T cells expressing CTTN_KR and individual cells tracked by ImageJ Fiji TrackMate plugin^28^^,^^29^ (n = 50 cells). (K) Total number of lamellipodia per cell, per 4-s time points in LifeAct-mScarlet expressing Jurkat T cells upon control and acetate treatment (n = 10 cells). (L) Representative still images of control and acetate treated LifeAct-mScarlet expressing Jurkat T cells at 0, 16, 32, 48, and 60^th^ seconds (for full Video see Video S3). Scale bar = 10 μm. (M) Quantification of western blot analysis of the level of acetylated cortactin in the splenocytes of alcohol-fed mice compared to water-fed control (n = 4 control mice, n = 3 alcohol-fed mice [EtOH] for blot images see Figure S3C). (N) Migrated cell count in an *in vitro trans*-well migration assay of primary cortactin knockout (CTTN KO) mouse CD4^+^ T cells compared to wild-type CD4^+^ T cells, counted by flow cytometry. Representative data shown from either one of three independent experiments (C, E, F, M, N) or one of two independent experiments (A, B, D, H, K, I, J, L) or three independent experiments combined (G) and expressed as mean ± SD, except for panels I and J where data are expressed as mean ± SEM. Statistical difference was determined by Student’s two-tailed t-test (A, B, D, G, M, N), one-way ANOVA (C, E, I, J) and area under the curve analysis (K). ∗p < 0.05; ∗∗p < 0.01; ∗∗∗p < 0.001; ∗∗∗∗p < 0.0001.

A recent review article by Mu et al. summarized the acetylation of various components of the cytoskeleton.^9^ We identified cortactin, a facilitator and stabilizer of F-actin branching, as a likely candidate for further research as it has acetylation sites in each of its 6.5 repeat regions within the actin-filament binding domain (ABD).^9^ Initially, it was believed that T cells express hematopoietic homolog of cortactin, hematopoietic lineage cell-specific protein 1 (HS1).^33^ But later, cortactin was discovered in dendritic cells (DCs), macrophages, and lymphocytes.^34^ HS1, in comparison to cortactin, has 3.5 cortactin repeat regions and acetylation sites on only 2 of those repeats.^35^ In addition, cortactin is a direct target of HDAC6.^9^^,^^36^ As such, treatment of C57BL/6 mice with HDAC6 inhibitor reduced disease severity in an SLE preclinical model and akin to our findings with acetate-exposed CIA and T cell dependent vaccination mouse models.^13^^,^^14^^,^^17^ Cortactin acetylation leads to decreased binding to F-actin and subsequent F-actin branching.^36^ We first confirmed cortactin expression in mouse primary CD4^+^ T cells by RNA sequencing (Figure S3A). Acetate treatment of Jurkat T cells increased acetylation levels of cortactin as shown by western blotting (Figures 3A and S3B). We then identified cortactin protein with high confidence (protein score −10lgP-value 189.32) in Jurkat T cells by Mass Spectrometry (ESI MS/MS) and confirmed its acetylation, e.g., at the c-terminal lysine 181 of peptide VDKSAVGFDYQGK (score −10logP 28.34). Evidence of cortactin acetylation by proteomics, compelled us to mutate lysine residues relevant for cortactin-F-actin binding. As such, we mutated 6 lysine residues, acetylation of which previously have been reported to inhibit binding of cortactin to F-actin, to arginine residues at the acetylation sites (CTTN_KR) within cortactin ABD (see Figure S4C for overexpression western blot analysis).^36^ Zhang et al., have demonstrated that the mutation of lysine residues to arginine blocks acetylation but still allows binding of cortactin to F-actin.^36^ Microscopy analysis of F-actin MFI in Jurkat T cells overexpressing CTTN_KR exhibited increased F-actin amounts even in the presence of acetate (Figure 3B). Further analysis of F-actin-bound cortactin by Förster resonance energy transfer (FRET) revealed reduced F-actin-bound cortactin levels upon acetate treatment, and that Jurkat T cells expressing mutated cortactin (CTTN_KR) were resistant to such reduction (Figures 3C and 3F). Further, we also quantified acetate’s effect on cortactin-F-actin binding in HEK293T cells and found similar results (Figure 3D). Next, in *trans*-well migration assays we found that the expression of acetylation-resistant cortactin (CTTN_KR) rescued acetate-induced migration deficiency (Figure 3E). To rule out the effect of CTTN_KR overexpression, we generated Jurkat T cells overexpressing wild type cortactin (Figures S4A and S4B). Simultaneous assessment of acetate’s effect on F-actin levels in Jurkat T cells overexpressing wild-type or CTTN_KR mutant cortactin revealed decreased F-actin in case of wild type cortactin expressing cells but not the mutant (Figures S4C–S4E). In addition, acetate exposure of Jurkat T cells overexpressing wild type cortactin led to decreased migratory capacity (Figure S4F). To recreate the 3-dimensional (3D) environment in which T cells migrate and function, we incorporated Jurkat T cells into Matrigel domes and imaged using Leica Thunder 3D Imaging microscopy (Videos S1 and S2, control and acetate treated, respectively). We observed decreased total distance migrated of acetate treated Jurkat T cells (Figure 3G). To better understand deficiencies in migration, we performed 2D Jurkat T cell tracking in cell culture treated wells by live-cell microscopy (Figure 3H). We found that acetate treated Jurkat T cells had a reduced linearity of forward progression and directional change rate, and that cells expressing acetylationresistant cortactin (CTTN_KR) were resistant to the effects of acetate (Figures 3I and 3J). Modifications to F-actin are required for lamellipodia formation and proper T cell migration.^3^ Cortactin has been shown to be required for lamellipodial persistence and cell migration.^37^ To study the highly dynamic lamellipodia formation and persistence, we generated Jurkat T cells expressing LifeAct-mScarlet-i_N1, a short peptide that binds F-actin and allows for visualization by confocal microscopy. Analysis of lamellipodia numbers, per cell, for the period of 60 s, revealed reduced lamellipodia formation upon acetate treatment of Jurkat T cells (Figures 3K, 3L, and Video S3). Finally, western blot analysis of the splenocytes isolated from alcohol-fed mice exhibited increased levels of acetylated cortactin in comparison to water-fed control mice (Figures 3M and S3C). In a proof-of-concept experiment, primary CD4^+^ T cells isolated from cortactin knockout mice exhibited severe impairment of migration in an *in vitro trans*-well assay, exhibiting little known importance of cortactin for proper T cell migration (Figure 3N). These findings indicate that alcohol consumption can directly increase acetylation of cortactin in secondary lymphoid organs (SLOs) such as spleen, and such an increase in T cells impairs T cell migration capacity.

Video S1. Tracking of control treated Jurkat T cell migration within 3D Matrigel Domes by Leica Thunder 3D imager, related to Figure 3G

Video S2. Tracking of acetate treated Jurkat T cell migration within 3D Matrigel Domes by Leica Thunder 3D imager, related to Figure 3G

Video S3. Jurkat T cells exposed to acetate exhibit reduced lamellipodia formation, related to Figures 3K and 3L Jurkat T cells expressing LifeAct-mScarlet treated with 5 mM acetate or vehicle (R10 medium) in R10 medium (n = 10 cells per treatment), visualized at 6 frames per second (each frame = 4 s) for 60 s.

### Acetate-induced F-actin deficiency impairs T cell activation and proliferation

**Figure 4:**
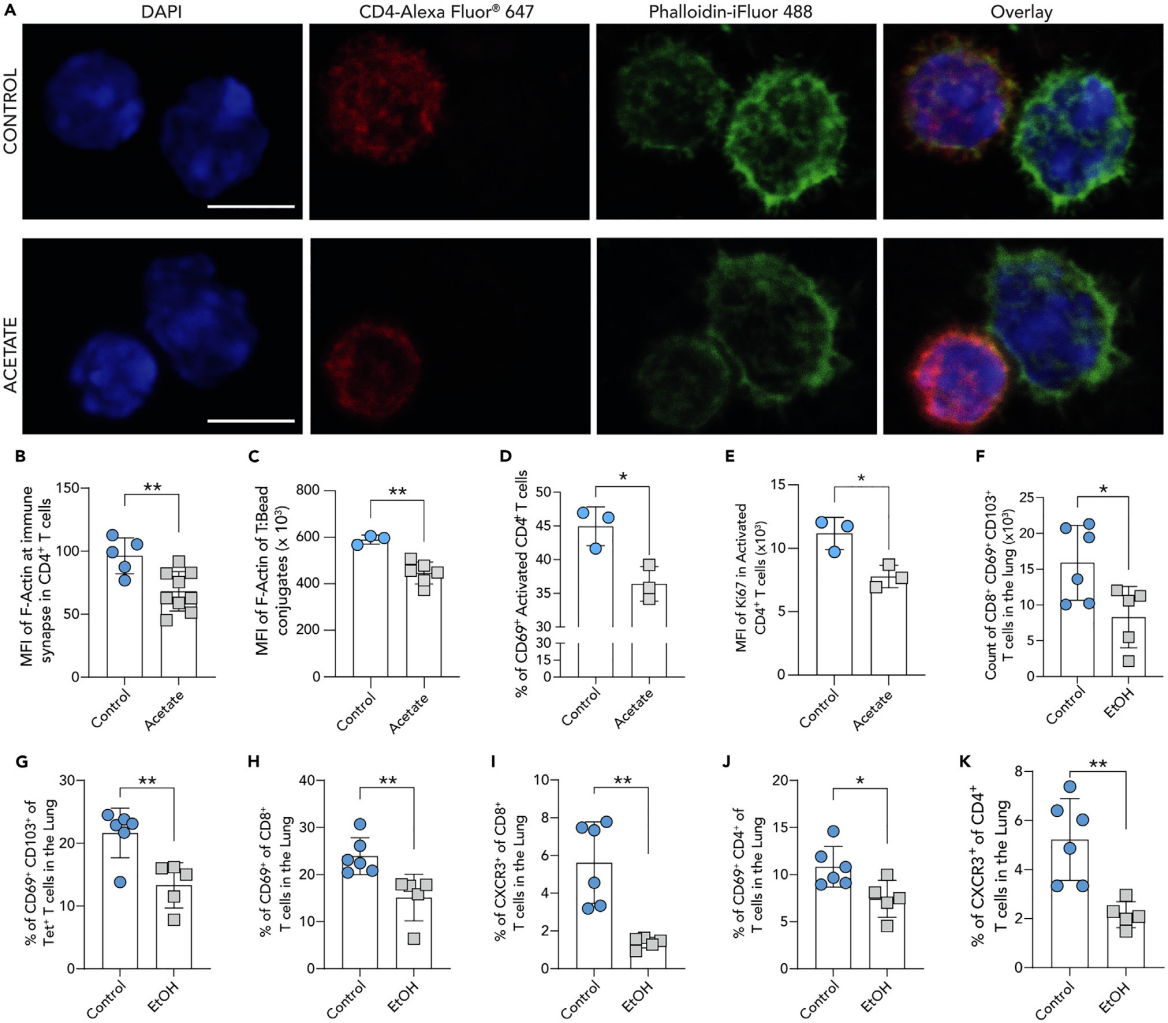
Acetate-induced F-actin deficiency reduces T cell activation and proliferation (A and B) Representative images of confocal laser scanning microscopy of T-DC cell conjugates for F-actin accumulation at the immune synapse, nuclei colored in blue by DAPI, F-actin colored in green by phalloidin-iFluor 488, and T cells colored in red by anti-CD4 antibody Alexa Fluor 647. Scale bar = 5 μm. (A) MFI of F-actin accumulated in CD4^+^ T cells at the immune synapse with NP-CGG pulsed DCs quantified by confocal laser scanning microscopy and (B) with mouse CD4^+^ T cell activator beads quantified by flow cytometry (for gating see Figure S5A). (C–G) Flow cytometry analysis of percentage of CD69^+^ CD25^+^ CD4^+^ and (D) Ki67 MFI of CD4^+^ T cells *in vitro* co-culture with NP-CGG pulsed DCs in the presence or absence of 5 mM acetate (for gating see Figure S5B). Flow cytometry analysis of CD69^+^ CD103^+^ (F) CD8^+^ T cells and (G) influenza specific tetramer (Tet^+^) CD8^+^ T cells in the lungs of alcohol-fed mice in comparison to water-fed control mice. (H and I) Flow cytometry analysis of percentage of CD69^+^ and (I) percentage of CXCR3^+^ of CD8^+^ T cells in the lungs of alcohol-fed mice in comparison to water-fed control mice. (J and K) Flow cytometry analysis of percentage of CD69^+^ and (K) percentage of CXCR3^+^ of CD4^+^ T cells in the lungs of alcohol-fed mice in comparison to water-fed control mice (each point represents one mouse. n = 6 control, n = 5 alcohol-fed mice) (for gating strategies of panels F–K see Figure S6). Representative data shown either from one of three independent experiments (D, E), or two independent experiments (A, B, C, F–K) and expressed as mean ± SD. Statistical difference was determined by Student’s two-tailed t-test. ∗p < 0.05; ∗∗p < 0.01; ∗∗∗p < 0.001; ∗∗∗∗p < 0.0001.

F-actin cytoskeleton is required for T cell—antigen presenting cell (APC) interaction and immune synapse formation.^38^^,^^39^^,^^40^ We performed confocal fluorescence microscopy to visualize F-actin at the immune synapse by phalloidin staining and quantified MFI values. For this, we isolated DCs and CD4^+^ T cells from previously NP-CGG immunized mice and incubated both cell types at 1:1 ratio, at 10^7^ cells per mL concentration (total volume 50 μL) following NP-CGG pulse of DCs and treatment of T cells (Figure 4A). We found a decrease in F-actin accumulation at the immune synapse within T cells between T cells and DCs (Figure 4B). To rule out the possible effect of acetate on DC immune synapse engagement, we repeated this experiment by using T cell activation beads. Activation of naive CD4^+^ T cells with anti-CD3 and anti-CD28 antibody coated beads in the presence of acetate also resulted in reduced F-actin amounts, as quantified by Alexa Fluor 488-phalloidin MFI (Figures 4C and S5A). Next, we performed co-culturing experiment of NP-CGG antigen-pulsed DCs and CD4^+^ T cells to better interrogate if acetate-directed F-actin deficiency affects T cell activation. T cells were either pre-treated with 5 mM acetate or vehicle, while antigen-pulse of DCs with NP-CGG was performed in an absence of acetate. Later upon combining DCs and T cells, acetate treatment was continued in an effort to replicate *in vivo* acetate exposure within lymphoid organs. Here, flow cytometry analysis revealed reduced surface CD69 levels on acetate treated CD4^+^ CD25^+^ T cells indicating reduced activation (Figures 4D and S5B). In the same experiment, flow cytometry analysis of acetate treated CD4^+^ T cells revealed that Ki67 levels, a proliferation marker, were reduced (Figures 4B and S5B). These experiments document the sensitivity of T cells to cytoskeletal deficiencies for migration, immune synapse formation, and activation, and they shed light on our previously published finding of reduced T_FH_-B cell conjugate formation upon acetate exposure *in vivo* and *in vitro.*^17^

### Acetate-exposed mice exhibit inhibited T cell responses in the lungs upon influenza infection

Since we have witnessed acetate’s effect on T cell cytoskeleton, we wondered whether alcohol-sourced acetate will impair T cell migration and activation during viral infection. For this, we utilized mouse influenza infection model. Here, alcohol consuming mice were infected with influenza H1N1 and two weeks later lungs isolated and analyzed. Flow cytometry analysis revealed reduced numbers of CD8^+^ CD69^+^ CD103^+^ resident T cells in the lungs of alcohol consuming influenza infected mice in comparison to water-fed control mice (Figures 4F and S6). Interestingly, we also found decreased percentage of CD69^+^ Tet^+^ influenza specific CD8^+^ T cells and CD69^+^ CD8^+^ T cells in alcohol consuming influenza infected mice in contrast to water-fed control mice (Figures 4G and 4H). In addition, we observed reduced percentage of CXCR3^+^ CD8^+^ T cells infiltrating the lungs of alcohol consuming mice in comparison with water-fed control mice (Figure 4I). Flow cytometry analysis of the CD4^+^ T cells in the lungs, revealed a reduction in percentage of CD69^+^ and also CXCR3^+^ CD4^+^ T cells in alcohol consuming mice in contrast to water-fed control mice (Figures 4J and 4K). Together these data reflect deficiencies in T cell migration, activation, and function *in vivo* and support our earlier adoptive transfer experiments of acetate-treated T cells.

## Discussion

**Figure 5:**
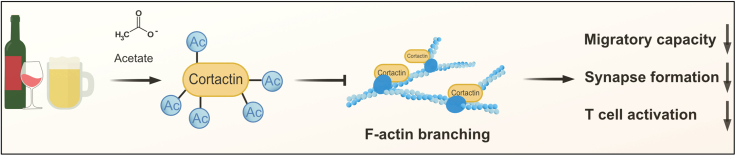
Overview of the mode of acetate’s action on T cell F-actin reorganization Alcohol consumption results in accumulation of acetate in lymphoid organs, increases acetylated cortactin levels in T cells. This results in reduced F-actin branching and consequently reduced migratory capacity, synapse formation, and activation.

Our findings indicate that alcohol-sourced acetate accumulates in lymphoid organs, increases cellular acetylated cortactin levels preventing F-actin branching and impeding T cell migration (overview Figure 5). The Arp2/3 complex is a part of F-actin branching nucleator complex.^40^ But it does require cortactin to facilitate its binding to F-actin. Whereas cortactin was shown to increase the affinity of Arp2/3 complex for F-actin by almost 20-fold.^41^ In addition, cortactin plays a key role in linking cortical F-actin to the cell membrane.^42^ Inhibition of Arp2/3 complex formation reduced T cell deceleration upon encountering high affinity antigens to a level observed for low affinity antigens.^43^ This finding potentially explains the weak immune synapse formation in our current study and also reduced T_FH_-B cell conjugate formation upon acetate exposure in our previously published findings.^17^ Currently, there are over dozen nucleating factors known to facilitate Arp2/3 complex binding to F-actin.^44^ Albeit, how each and every one of such nucleating factors, including cortactin, play a role in T cells is poorly understood. F-actin rearrangement enables cells to migrate, direct adhesion molecules to cell-cell contact zones, divide, and regulate various other cellular processes.^45^^,^^46^ Cytoskeleton is one of the central elements for optimum T cell function.^47^ For example, functional actin cytoskeleton has been shown to be important for T cell sampling of antigen-MHCII complexes on APCs with subsequent role in immune synapse formation.^38^ CD4^+^ and CD8^+^ T cell migration patterns through SLOs differ, CD4^+^ T cells spend more time probing MHCII molecules presented on DCs and are significantly faster in entering and exiting SLOs than CD8^+^ T cells.^48^^,^^49^^,^^50^ It is possible that diminished F-actin branching can therefore mitigate TCR coupling with high-affinity antigens and reduce T cell arrest and subsequent activation. Interestingly, in a mouse model of type 1 diabetes, mice fed with acetate-yielding chow exhibited a reduction in autoreactive T cells (both CD4^+^ and CD8^+^) and ultimately reduced the incidence of the disease.^51^ In another study of experimental autoimmune encephalomyelitis (EAE), cortactin knockout mice exhibited decreased incidence, disease severity, and infiltration of the central nervous system by CD4^+^ T cells.^52^ Interestingly, we observed the same effect in alcohol-fed EAE mice.^17^ Similarly, in an influenza infection model, alcohol-fed mice exhibited decreased homing to lungs, reduced CD69, CD103, and CXCR3 positive tissue resident CD8^+^ T cells. Tsuji et al., have previously shown the effect of HDAC6 inhibition, inadvertently increased cortactin acetylation, on CD8^+^ T cell activation deficiency.^53^ These findings go hand-in-hand with our findings in the context of influenza infection. Moreover, cortactin is overexpressed in B cells of chronic lymphoblastic leukemia patients (CLL), implicated in T cell acute lymphoblastic leukemia (T-ALL), and promotes migration in cancer.^54^^,^^55^ Identification of an Achille’s heal of T cell migration, motility, and immune synapse formation can set the stage for pharmacological and/or natural targeting in cases of autoimmunity. Further, such interventions can also help improve immune responses to vaccinations. In an evolutionary perspective, hominids adapted to alcohol metabolism by feeding on fermenting fruits from the forest floor.^56^ It is possible that indirect alcohol consumption mitigated adverse immune responses in early humans under high microbial load. Today, we see the same effects reflected in mitigation of autoimmunity and susceptibility to infections among alcohol consumers.^57^

### Limitations of the study

Although data shown herein directly link alcohol consumption to increased acetylation of cortactin, it is important to note that alcohol-sourced acetate can have other effects on T cell biology. While our study provides a robust result on the new role of cortactin in T cells, there are currently no mouse strains where cortactin is knocked out specifically in T cells, hence our limited use of *in vivo* models. Additionally, cortactin is highly conserved among different immune cells and tissues. Therefore, warranting future studies to measure the effect of cortactin acetylation on antigen presenting cells, B cells, macrophages, and other types of cells.

## STAR★Methods

### Key resources table

**Table:** 

REAGENT or RESOURCE	SOURCE	IDENTIFIER
**Antibodies**		

Rat Anti-mouse CD11b, PE/Cy7	BD Biosciences, San Jose, USA	Cat#552850; RRID: AB_394491
Rat Anti-mouse CD19, PE/Cy7	Biolegend, San Diego, USA	Cat#115520; RRID: AB_313655
Armenian Hamster Anti-mouse CD3ε, FITC	Biolegend, San Diego, USA	Cat#100306; RRID: AB_312671
Rat Anti-mouse CD4, PerCP/Cy5.5	Biolegend, San Diego, USA	Cat#100540; RRID: AB_893326
Rat Anti-mouse CD4, Alexa Fluor® 647	Biolegend, San Diego, USA	Cat#100424; RRID: AB_389324
Rat Anti-mouse CD3, BV510	Biolegend, San Diego, USA	Cat#100234; RRID: AB_2562555
Rat Anti-mouse CD4, FITC	Biolegend, San Diego, USA	Cat#100406; RRID: AB_312691
Rat Anti-mouse CD25, PE	Biolegend, San Diego, USA	Cat#102008; RRID: AB_312857
Rat Anti-mouse FOXP3, BV421	Biolegend, San Diego, USA	Cat#126419; RRID: AB_2565933
Rat Anti-human CD4, FITC	Biolegend, San Diego, USA	Cat#357405; RRID: AB_2562356
Rat Anti-mouse B220, FITC	Biolegend, San Diego, USA	Cat#103206; RRID: AB_312991
Mouse Anti-mouse β-actin, HRP linked	Abcam, Cambridge, United Kingdom	Cat#ab49900; RRID: AB_867494
Rat Anti-mouse CD8, FITC	BD Biosciences, San Jose, USA	Cat#553031; RRID: AB_394568
Rat Anti-mouse CD62L, APC	Biolegend, San Diego, USA	Cat#104412; RRID: AB_313099
Rat Anti-mouse CD44, PE	Biolegend, San Diego, USA	Cat#103008; RRID: AB_312959
Rat Anti-mouse CD45R/B220, Alexa Fluor 488	Biolegend, San Diego, USA	Cat#103225; RRID: AB_389308
Rabbit Anti-mouse Histone 3, unconjugated	Abcam, Cambridge, United Kingdom	Cat#ab1791; RRID: AB_302613
Rabbit Anti-mouse Histone 3 Lysine 27 Acetyl, unconjugated	Abcam, Cambridge, United Kingdom	Cat#ab4729; RRID: AB_2118291
Donkey Anti-rabbit IgG H&L, (HRP)	Abcam, Cambridge, United Kingdom	Cat#ab6802; RRID: AB_955445
Mouse Purified anti-Cortactin (CTTN) Antibody	Biolegend, San Diego, USA	Cat#868102; RRID: AB_2810812
Rabbit Anti-acetyl Cortactin Antibody	Merck, Darmstadt, Germany	Cat#09-881; RRID: AB_10584980
Goat anti-mouse IgG Alexa Fluor® 594	Biolegend, San Diego, USA	Cat#405326; RRID: AB_2563308
Armenian Hamster anti-mouse CD69 PerCP	Biolegend, San Diego, USA	Cat#104520; RRID: AB_940495

**Deposited data**		

Proteomics	ProteomeXchange	PXD037723
Gene Expression	Gene Expression Omnibus	GSE216415

**Experimental models: Cell lines**		

T cell leukemia JURKAT	Leibniz Institute DSMZ German Collection of Microorganisms and Cell Cultures GmbH, Braunschweig, Germany	Cat#ACC282
HEK293T	Leibniz Institute DSMZ German Collection of Microorganisms and Cell Cultures GmbH, Braunschweig, Germany	Cat#ACC635
b.End3	Merck, Darmstadt, Germany	Cat#96091929-1VL

**Experimental models: Organisms/strains**		

C57BL/6NCrl	Charles River	N/A
Cttn-/- C57BL/6J	Prof. Dr. Klemens Rottner from Helmholtz Centre for Infection Research (Braunschweig, Germany)	N/A

**Recombinant DNA**		

CTTN Lysine to Arginine mutant synthesized	Thermo Fisher	N/A

**Software and algorithms**		

TrackMate	Fiji Image J	N/A

### Resource availability

#### Lead contact

Further information and requests for resources and reagents should be directed to and fulfilled by the lead contact, Mario M. Zaiss (mario.zaiss@uk-erlangen.de).

#### Materials availability

This study did not generate new unique reagents.

### Experimental model and study participant details

#### Mice

Female, 6 weeks old C57BL/6NCrl mice were purchased from Charles River (Germany). Mice were co-housed for 3 weeks prior to start of experiments. Cortactin knock out mice were kindly provided by Prof. Dr. Klemens Rottner from Helmholtz Centre for Infection Research (Braunschweig, Germany). All mice were housed, and experiments were conducted under specific pathogen-free conditions. All protocols for animal experiments were approved by the local ethics authorities of the Regierung von Unterfranken (55.2-2532-2-703, 55.2.2-2532-2-1515).

### Method details

#### Adoptive transfer experiments

CD4^+^ T cells (naïve or total) were enriched with naïve or total CD4^+^ T cell negative enrichment kits (Stemcell, Biolegend) from the spleens of female, 8 weeks old C57BL/6NCrl mice. T cells were then either treated with 5 mM sodium acetate or sodium matched R10 medium (RPMI 1640, 10% v/v Fetal Bovine Serum, supplemented with 50 μM β-mercaptoethanol, non-essential amino acids, 2 mM L-Glutamine, sodium pyruvate, 10 mM HEPES, and 1% penicillin and streptomycin) for 4 hours. Cells were spun down (300xg) and washed two times with PBS at room temperature (RT). Cells were then loaded with CellTrace® cell dye (CFSE and Violet, for control and acetate treatment) according to manufacturer’s instructions (Thermo Fisher) and incubated further 30 minutes with corresponding treatment (control or acetate containing R10 medium). Cells were then counted and adjusted to 3x10^7^ cells for each treatment and combined to make 6x10^7^ cells in PBS prior to transfer to recipient mice (C57BL/6NCrl, female, matching age) intravenously in the tail vain. Recipient mice were sacrificed 2 hours and 48 hours post adoptive transfer, and spleens collected for flow cytometry.

#### NP-CGG immunizations

Female, 8 weeks old C57BL/6NCrl mice were co-housed and 1 week before immunization, mice were given either water, 10% (v/v) Ethanol (Roche) and 2% (w/v) Glucose (Sigma), all feedings were changed every 3 days. For primary NP-CGG immunization, mice were injected intraperitoneally (i.p.) with 100 μg of NP-CGG (LGC Biosearch Technologies) in 200 μL of alum (Thermo Scientific) according to manufacturer’s instructions. 14 days later, mice were boosted with 100 μg of NP-CGG in 200 μL of alum. One week later, mice were sacrificed, and spleens collected for isolation of CD4^+^ T cells and dendritic cells (Stemcell, Biolegend) for *in vitro* experiments.

#### Influenza infection model

Starting 1 week before infection, C57BL/6NCrl mice were given either 2% (w/v) Glucose water or 10% (v/v) Ethanol (Roche) and 2% (w/v) Glucose (Sigma). All feedings were changed every 3 days and were continued throughout the infection. Animals were infected by intranasal instillation of 200 PFU of H1N1 A/Puerto Rico/8/1934 in 50 μL PBS under general anesthesia. Lungs were harvested 14 days post infection. For T cell analyses, lungs were cut in pieces and were treated with 500 units Collagenase D and 160 units Dnase I in 2 mL R10 medium (RPMI 1640 supplemented with 10% FCS, 2 mM L-Glutamine, 10 mM HEPES, 50 μM β-mercaptoethanol and 1% penicillin/streptomycin) for 45 minutes at 37°C. Lungs were then mashed through a 70 μm cell strainer before subjected to erythrocyte lysis. One fifth of the cell suspension was stained with a pentamer for NP366-374-specific CD8^+^ T cells (1:40; ProImmune), anti-CD8-BV711 (1:300, clone 53-6.7, Biolegend), anti-CD4-BV605 (1:300, clone RM4-5, Biolegend), anti-CD45-BV510 (1:300, clone 30F11, Biolegend), anti-CD69-PerCP-Cy5.5 (1:200, clone H12F3, Biolegend), anti-CD103-PE (1:200, clone 2E7, Invitrogen), anti-CXCR3-APC-Fire750 (1:100, clone CXCR3-173, Biolegend), anti-CD127-FITC (1:300, clone A7R34, Biolegend), and anti-KLRG1-PE-Cy7 (1:300, clone 2F1, Invitrogen). Data were acquired on an AttuneNxt (ThermoFisher) and analysed using FlowJo™ software (Tree Star Inc.).

#### Flow cytometry

Spleens were smashed and filtered through 40 μm gauze (BD Biosciences). Single-cell suspensions were incubated with antibodies for 30 minutes at 4°C, washed and analyzed with Cytoflex S flow cytometer (Beckman Coulter). Flow cytometry analysis of *in vitro* experiments were performed by centrifuging cells in 96-well V-bottom plates at 300xg and 4°C for 5 minutes. Antibody staining for surface markers were performed at 4°C for 30 minutes, followed by two times wash with FACS Buffer (PBS, 2 mM EDTA, 2% Fetal Bovine Serum) and one-time wash with PBS. Cells were then resuspended in 100 μL of PBS and fixed with addition of 100 μL of IC Fixation Buffer (Thermo Fisher) and incubated at 4°C for 15 minutes protected from light. Cells were then washed three times with 1x Permeabilization buffer and incubated for 1 hour at RT for permeabilization (Thermo Fisher) followed by intracellular staining. Intracellular staining of filamentous actin was performed by incubating cells with phalloidin-Alexa-Fluor®-488 (Abcam) at 1:1000 dilution for 2 hours at RT in PBS with 1% w/v BSA, protected from light.

#### *In vitro* migration

Jurkat T cells or naïve CD4^+^ T cells were treated with 5 mM sodium acetate containing R10 medium for 4 hours prior to Transwell migration assay. T cells were then seeded at 10^5^ cells in 100 μL total volume above 3 μm and 8 μm pore size transwell inserts (GreinerBio), mouse CD4^+^ T cells and Jurkat T cells, respectively. Transwell inserts were placed in 24-well plate containing R10 medium matching the volume height within the insert (600 μL), 20 ng/mL of CCL19 (mouse CD4^+^ T cells) or 5 ng/mL CCL2 (Jurkat T) added to the bottom well and incubated for 4 hours. Migrated cells were collected from the bottom wells and centrifuged, resuspended in 100 μl FACS buffer, quantified by flow cytometry. In case of primary CD4^+^ T cells, we have also performed trans-well migration assay through b.End3 endothelial cell barrier.

#### *In vitro* co-culture of antigen-presenting cells (APCs) and T cells

Dendritic cells (DCs) and CD4^+^ T cells were enriched from C57BL/6CNrl mice immunized two times, 14 days apart, with 100 μg NP-CGG in 200 μL of Imject™ Alum and sacrificed on day 21 (Stemcell, Thermo Fisher, LGC). CD4^+^ T cells were pre-treated with 0.5, 2.0, or 5 mM sodium acetate or vehicle (R10 medium) containing R10 medium for 4 hours. Meanwhile, DCs were pulsed with 20 μg/mL NP-CGG (LGC) and 100 ng/mL LPS (Sigma Aldrich) in R10 medium for 4 hours. CD4^+^ T cells were adjusted to 10^5^ cells per well of 96-well flat bottom cell culture plate. DCs were then washed two times with fresh medium and combined with CD4^+^ T cells for 48 hours. Final total volume per well was 200 μL. At the conclusion of 48-hour incubation, cells were moved to a 96-well V-bottom cell culture plate, wells flushed and collected. Cells were fixed and stained as explained under Flow Cytometry section.

#### *In vitro* activation of CD4^+^ T cells

Naïve CD4^+^ T cells were enriched from C57BL/6CNrl mice (Stemcell). Then, the cells were pre-treated with 0.5, 2.0, or 5 mM sodium acetate or vehicle (R10 medium) containing R10 medium for 4 hours. Dynabeads™ Mouse T-Activator CD3/CD28 beads were added according to manufacturer’s instructions for 4 hours (Thermo Fisher). At the conclusion of 4 hours, cells were centrifuged at 4°C at 200xg for 10 minutes and fixed with IC fixation buffer (Thermo Fisher) in 200 μL (100 μL IC Fixation buffer + 100 μL PBS) for 15 minutes at 4°C. Following fixation, cells were washed with PBS three times and permeabilized with 1x eBiosciences™ permeabilization buffer for 1 hour at room temperature (Thermo Fisher). Phalloidin-iFluor 488 (Abcam) stock solution was diluted in PBS containing 1% w/v BSA 1000x. After permeabilization, cells were washed three times and Phalloidin-iFluor 488 solution added for 2 hours at RT.

#### RNA sequencing

Naïve CD4^+^ T cells were treated with 2.0 mM sodium acetate or vehicle (R10 medium) containing R10 medium for 4 hours. RNA was isolated using the Rneasy micro kit (Qiagen). Sequencing was performed on the Illumina platform (Novogene, Europe). Raw reads were processed through fastp (Galaxy). Mapping the processed data to the reference genome (GRCm39) was performed using Star software, and alignment visualization was done with the integrative genomics viewer (IGV). Raw data is available under GEO accession GSE216415.

#### Immune synapse formation

Dendritic cells (DCs) and CD4^+^ T cells were enriched from C57BL/6CNrl mice immunized two times, 14 days apart, with 100 μg NP-CGG in 200 μL of Imject™ Alum and sacrificed on day 21 (Stemcell, Thermo Fisher, LGC). CD4^+^ T cells were pre-treated with 0.5, 2.0, 5.0 mM acetate or vehicle (R10 medium) for 6 hours in R10 medium. Simultaneously, DCs were pulsed with 20 μg/mL NP-CGG in R10 medium for 6 hours. Then, 10^6^ pre-treated naïve CD4^+^ T cells and 10^6^ NP-CGG pulsed DCs were combined in 100 μL of R10 medium for 15 minutes at 37°C. Then 1.5 mL of 1.5% paraformaldehyde (PFA) was added to arrest T-DC conjugates. Cells were then washed with PBS+2% FCS (v/v) three times. Cells were then stained for flow cytometry with anti-mouse CD4 BV421™ and anti-mouse CD11c BV510™, or for microscopy anti-CD4 Alexa Fluor 647™ only in PBS+2% FCS (v/v) for 30 minutes at 4°C. Cells were washed, and fixed with eBiosciences™ IC fixation buffer for 15 minutes at 4°C. Cells were washed with PBS three times and permeabilized with 1x eBiosciences™ permeabilization buffer for 1 hour at room temperature. Phalloidin-iFluor 488 stock solution was diluted in PBS containing 1% w/v BSA 1000x. After permeabilization, cells were washed three times and Phalloidin-iFluor 488 solution added for 2 hours at RT. Then, cells were washed with PBS three times with 5-minute incubations in PBS to remove all unbound Phalloidin-iFluor 488. For microscopy samples, nuclei were staining with DAPI in PBS for 5 minutes and then washed and resuspended in 60 μL of PBS. Drops of 30 μL were placed on microscope slides and allowed to dry in the dark. After, ProLong™ Glass Anti-fade mounting medium was used to mount slides and let cure for 24 hours. Samples for flow cytometry were resuspended after washing Phalloidin-iFluor 488 out and resuspending in PBS.

#### Förster Resonance Energy Transfer (FRET) experiment

For this, 2x10^4^ control or 5mM acetate treated Jurkat T cells were seeded onto poly-L-lysine coated cover slips (0.01% poly-L-lysine (Sigma) for 15 minutes, followed by 3x PBS wash). After cells settled and attached (1 hour at 37° with 5% CO_2_), medium was removed, and cells fixed with 1.5% PFA solution at RT for 20 minutes. After a 3x PBS wash, cells were permeabilized with 1x eBiosciences™ permeabilization buffer for 1 hour at room temperature. After permeabilization, cells were washed three times. Samples were blocked with PBS+0.5% w/v BSA for 30 minutes at RT. Anti-cortactin primary antibody (Biolegend, clone 4F11) was diluted in PBS+0.5% w/v BSA and incubated with cells at RT for 1 hour. Cells were gently washed 5x with PBS. Goat anti-mouse IgG AF594 was diluted in PBS+0.5% w/v BSA and incubated with cells for another 1 hour at RT. Following, cover slips were gently washed with PBS 3x. Phalloidin-iFluor 488 stock solution was diluted in PBS containing 0.5% w/v BSA 1000x. Phalloidin-iFluor 488 solution added for 2 hours at RT. Then, cells were washed with PBS three times with 5-minute incubations in PBS to remove all unbound Phalloidin-iFluor 488. For microscopy samples, nuclei were staining with DAPI in PBS for 5 minutes and then washed and resuspended in 60 μL of PBS. Drops of 30 μL were placed on microscope slides and allowed to dry in the dark. After, ProLong™ Glass Anti-fade mounting medium was used to mount slides and let cure for 24 hours. Images were taken with Leica Stellaris 8 Broadband WLL & Tau-Sense LSM at Optical Imaging Centre Erlangen core facility with 100x NA 1.44 objective. Cells were first focused on by visualizing nuclei, then images captured only with the excitation at 488nm, and signals captured at bandpass set for iFluor 488 and Alexa Fluor™ 594 simultaneously. Controls, no iFluor 488, and no primary anti cortactin were used to confirm energy transfer specificity.

#### T cell migration within 3D Matrigel™

Jurkat T expressing mScarlet-H cells were maintained in R10 medium at a denstity of 1x10^5^-1x10^6^ cells/ml. Jurkat T cells were then split into two groups of untreated and treated cells. Treatment of Jurkat T cells was performed with 5 mM sodium acetate containing R10 medium for a total of 48 hours. Afterwards, approximately 1.5x10^5^ cells were spun down, supernatant was aspirated, and cells were resuspended in 25 μL ice cold R10 medium. Then, cells were mixed with 25 μL Matrigel (cold tips) and a dome was pipetted in the middle of a pre-warmed 8-well slide (Ibidi). The slide was then placed in an incubator to let the Matrigel polymerize and after 15 minutes the slide was taken out, and the chambers were filled with 280 μl of warm R10 medium. Additionally, 5 mM acetate was added to the chambers containing acetate-treated Jurkat T cells. Excitation was carried out at 555 nm and emission detected with 572 nm filter, timelapses with approximately 100 μm Z-Stack were acquired for 3-4 hours with Leica Thunder Imager with 20x dry objective with NA 0.8 and instant computational clearing. Timelapses were analyzed with TrackMate plugin from ImageJ software and visualized using VisualizeTracks plugin.^58^

#### Live cell tracking

Jurkat T cells and Jurkat T cells overexpressing acetylation resistant cortactin (CTTN_KR) were cultured in R10 medium supplemented with 5 mM sodium acetate for 4 hours. For acetate treatment control, Jurkat T cells were cultured in R10 medium. As a control for plasmid electroporation, Jurkat T cells electroporated with mScarlet_H plasmid were used. The cells were then transferred to incubated and CO2 gassed Zeiss Cell Discoverer (Carl Zeiss) live imaging microscope. Then images were taken by brightfield illumination with Apochromat 10x objective in 2-minute intervals for a total of 180 minutes. Cells were then tracked using TrackMate plugin for Fiji.^58^^,^^59^ To visualize individual tracks, tracks were obtained from TrackMate and re-centered at the origin of the plot.

#### Live cell imaging of lamellipodia

Jurkat T cells overexpressing pLifeAct_mScarlet-i_N1 (see Electroporation methods), were treated with 5 mM sodium acetate or vehicle (R10 medium) containing R10 medium for 4 hours. Later, cells were seeded into μ-Slide 8 well microscope slides (ibidi) and placed into incubated microscopy chamber where 5 ng/mL concentration of CCL2 was added to induce polarization. Zeiss Spinning Disc Axio observer Z1 microscope with oil immersed 63x 1.2 NA objective and EVOLVE 512 EMCCD with Yokogawa CSU-X1M 5000 was used to locate cells. Cells were illuminated with a laser at 561 nm wavelength, and emissions captured through band-pass filter 629/62 every 4 seconds for 60 seconds. Images were later processed and analyzed for numbers of lamellipodia at each time point and counted. Statistical analysis was done by area under the curve analysis.

#### Cortactin lysine to arginine mutagenesis

Recombinant cortactin gene with lysine residues 87, 124, 161, 198, 235, 272, 309, and 319 replaced with arginine, was synthesized, and cloned into pcDNA3.1plus vector by GeneArt™ Thermo Fisher. Jurkat T cells were electroporated as described below and clones were selected with 800 μg/mL G418 (InvivoGen) antibiotic for 7 passages.

#### Electroporation of Jurkat T cells

Jurkat T cells were maintained in R10 medium. 10^7^ Jurkat T cells were washed two times with RPMI1640 medium only, centrifugation steps at 200xg for 10 minutes, at RT. Jurkat T cells were then washed one more time with 5 mL of OptiMEM® medium and resuspended in 100 μL of OptiMEM® medium. 25 μg (1 μg/μL concentration) of plasmid DNA was added to electroporation 4 mm cuvettes (Bio-Rad) first and then cells added to the cuvettes. Electroporation was carried out with Bio-Rad Gene Pulser Xcell Electroporation Systems at Square wave, 500 Volts, 3 ms, Pulse 1, Interval 0). After the electroporation, cells were transferred to T25 cell culture flasks with 5 mL of prewarmed (37°C) R10 medium and incubated overnight. For control purposes, no plasmid DNA containing cell mixture was used for electroporation.

#### Real-time deformability cytometry (RT-DC)

RT-DC measurements were performed using an AcCellerator instrument (Zellmechanik Dresden). T cells were resuspended in measurement buffer composed of 0.6% (m/v) methyl cellulose dissolved in phosphate-buffered saline adjusted to a viscosity of 60 mPa s at 24°C using a falling ball viscometer (Haake; Thermo Fisher Scientific). The suspension of T cells was loaded into a 1 mL syringe attached to a syringe pump and connected by PEEK-tubing (IDEX Health & Science) to a microfluidic chip made of PDMS bonded on cover glass. A second syringe with pure measurement buffer was attached to the chip. The microfluidic chip consisted of two inlets and one outlet. The measurement was performed in a narrow channel constriction of 15 × 15 μm square cross section. The total flow rate was 0.024 μL/s, with sheath flow rate of 0.018 μL/s and sample flow rate of 0.006 μL/s. Measurement temperature was 28°C. Images were acquired at a frame rate of 1250 fps. Cells were detected in a region of interest of 250 × 80 pixels. The cell images were analyzed using the analysis software Shape-Out version 2.3.0 (available at https://github.com/ZELLMECHANIK-DRESDEN/ShapeOut2) and Python 3.7 using dclab library. We applied a gate for cross-sectional cell size (15-35 μm^2^) and for the area ratio (1–1.05). The calculation of deformation, a measure of how much the cell shape deviates from circularity, was obtained from the image using the projected area and cell contour length calculated from the convex hull. Statistical analyses were carried out using a one-dimensional linear mixed model that incorporates fixed effect parameters and random effects to analyze differences between cell subsets and replicate variances, respectively. P-values were determined by a likelihood ratio test, comparing the full model with a model lacking the fixed effect term.

#### Protein isolation and western blot

After acetate treatment of Jurkat T cells, naïve CD4^+^ T cells activated either with 0.5 μg/mL PMA, 1 μg/mL ionomycin or NP-CGG-pulsed CD11c+ DCs then depleted off DCs, T cells were washed two times with ice-cold PBS containing 5 mM sodium butyrate, and nuclear/cytoplasmic protein was extracted according to manufacturer’s instructions (Thermo Fisher). Protein fractions were then frozen and stored at -80°C until analyzed by western blotting. Protein extracts were separated on 4-12% Bis-Tris gradient SDS-polyacrylamide gel, then transferred onto PVDF membrane, blocked with 5% milk in TBS 0.05% Tween 20 for 1 hour. Then membranes were probed with anti-cortactin, anti-acetyl cortactin, anti-GAPDH, anti-beta-actin, anti-histone 3, anti-acetyl-lysine-27-histone-3, and anti-histone 3 antibodies, and visualized with appropriate HRP-conjugated secondary antibody. The signal acquired from chemiluminescence (Celvin S) for protein of interests were optimized to loading control and quantified with Image J blot analysis tool.

#### Proteomic analysis of cortactin

For proteomic samples containing about 2 mg of total protein, Jurkat T cells were treated either with 5mM acetate in R10 medium or without acetate as the control. Cells were washed three times with ice cold PBS, lysed with sonication, and cleared by Ultracentrifugation at 45000 x*g*. Mass spectrometry (ESI/LC/MS/MS) was performed as described previously.^60^ In short, peptides for a topdown proteomic approach were prepared applying filter-aided sample preparation (FASP).^61^ After reduction and alkylation on a 10kDa membrane filter a total of 1.5 μg of protein of each sample was digested with 1μg trypsin for 18h at 37°C. Resulting peptides were extracted by centrifugation and desalted on C18 stage tips. Prior to MS analysis, tryptic peptides were dried under vacuum and resuspended in 15μl of 10% formic acid (FA). MS analysis was carried out as described.^60^ In short, resulting peptides (niport. 1 μg) were loaded on a nanoflow Ultimate 3000 HPLC (Dionex, Sunnyvale, CA, USA) for separation on an EASY-Spray column (Thermo Fisher Scientific; C18 with 2 μm particle size, 50 cm × 75 μm), with a flow rate of 200 nL/min by increasing acetonitrile concentrations over 120 min. All samples were analyzed on an Orbitrap fusion (Thermo Fisher Scientific, Waltham, MA, USA) with the previously described MS/MS settings.^60^ ESI spray voltage was 2.0 kV, scan range 300–2000 (*m*/*z*), with a maximum injection time of 50 ms and an AGC target of 400 k for the first stage of mass analysis (MS^1^). The most intense ions were selected for collision-induced dissociation with a collision energy of 35%, a maximum injection time of 250 ms, and an AGC target of 100 for the second stage of mass analysis (MS^2^). All raw files were analyzed using PEAKS Studio 8.5 (Bioinformatics Solutions, Waterloo, Ontario, Canada;^62^) and searches were performed against the *Homo sapiens* niport database downloaded in July 2019. Oxidation of methionine and acetylation of lysin were set as dynamic modifications and carbamidomethylation of cysteines as static modification, Parent mass tolerance was set to 10.0 ppm and fragment mass tolerance to 0.5 Da for tryptic peptides allowing a maximum of two missed cleavages. The MS proteomics data will be deposited to the ProteomeXchange Consortium via the PRIDE^63^ partner repository with the dataset identifier PXD037723.

#### Histone extraction

Naïve CD4^+^ T cells were pre-treated with 0.5, 2.0, 5.0 mM sodium acetate or vehicle (R10 medium) containing R10 medium for 4 hours. Cells were then washed with ice cold PBS supplemented with 5 mM sodium butyrate to preserve histone acetylation. Then the cytoplasm was extracted by resuspending cells in ice cold extraction buffer at 10^7^ cell/mL, incubating on ice for 5 minutes, and centrifuging at 6500xg for 10 minutes at 4°C. Supernatant was discarded and the cytoplasm extracted for the second time. Histones were extracted by resuspending the pellet in 0.25 M HCl at a density of 4x10^7^ cells/mL. Samples were then sonicated for 30 seconds, placed on ice, then sonicated for further 30 seconds. Afterwards, tubes were placed on rollers at 4°C for 1 hour. Then, samples were centrifuged at 12000xg for 10 minutes at 4°C. Supernatant was collected and neutralized with 2M NaOH at 1/10^th^ of the volume of the supernatant. Extracted histones were frozen and stored at -80°C until analyzed by western blotting.

#### Protein isolation from splenocytes

Spleens of alcohol-fed and water-fed C57BL/6NCrl mice were isolated and placed in ice cold PBS containing 5 mM sodium butyrate after 3 weeks of feeding at steady-state. Spleens were smashed and filtered through 40 μm gauze (BD Biosciences). Single-cell suspensions were spun down and resuspended in 5 mL of 1x Red Blood Cell Lysis buffer (Biolegend) and incubated on ice for 5 minutes with occasional swirl. Then, 25 mL of ice-cold PBS was added, and cells centrifuged at 300xg for 10 minutes. Cells were resuspended in 10 mL ice-cold PBS and spun down. Protein isolation of cytoplasmic and nuclear fraction was performed with NE-PER™ Nuclear Cytoplasmic Extraction (Thermo Fisher) supplemented with Pierce™ Protease inhibitor cocktail tablet (Thermo Fisher). Isolated proteins were kept at -80°C until analyzed by western blotting.

#### ^13^C citrate quantification

Label incorporation into citrate was analyzed by gas chromatography (GC – MS). In brief, cells were subjected to methanol-chloroform-water extraction and polar phase was dried under vacuum. For derivatization, 20 μL per sample of 40 mg methoxamine per mL pyridine were added and incubated at 30°C for 90 minutes, followed by the addition of 80 μL per sample of MSTFA, incubated at 37°C for 60 minutes. GC-MS analysis was performed on an Agilent 7890B GC – Pegasus 4D-C GCxGC TOF MS, coupled to a Gerstel autosampler. Samples were injected in splitless mode (injection volume 1 μL). The following temperature program was applied during sample injection: initial temperature of 80°C for 3 seconds followed by a ramp of 7°C/seconds to 210°C and final hold for 3 minutes. Gas chromatographic separation was performed with a Rxi-5MS column (30 m length, 250 μm inner diameter, 0.25 μm film thickness (Restek). Helium was used as carrier gas with a flow rate of 1.2 mL/minute. Gas chromatographic separation was performed with the following temperature gradient: 2 minutes initial hold at 68°C, first temperature gradient with 5°C/minute up to 120°C, second temperature gradient with 7°C/minute up to 200°C, and a third temperature gradient with 12°C/minute up to 320°C with a final hold for 6 minutes. The spectra were recorded in a mass range of 60 to 600 m/z with an acquisition rate of 20 spectra/second. Data was analyzed with ChromaTOF (Leco) and MetMax software.^64^

#### Short-chain fatty acid (SCFA) measurements

4-5 biological replicates of each treatment (control or ethanol feeding) mouse spleens were placed directly into diethyl ether and weighed in a 2 mL polypropylene tube. The tubes were kept in a cool rack throughout the extraction. 50 μL 33% HCl was added, and samples were vortexed for 1 minute and centrifuged for 3 minutes at 4°C. The organic phase was transferred into a 2 mL gas chromatography (GC) vial. For the calibration curve, 100 μl of SCFA calibration standards (Sigma) were dissolved in water to concentrations of 0, 0.5, 1, 5 and 10 mM and then subjected to the same extraction procedure as the samples. For GCMS analysis 1 μl of the sample (4-5 replicates) was injected with a split ratio of 20:1 on a Famewax, 30 m x 0,25 mm iD, 0.25 μm df capillary column (Restek). The GC-MS system consisted of GCMS QP2010Plus gas chromatograph/ mass spectrometer coupled with an AOC20S autosampler and an AOC20i auto injector (Shimadzu). Injection temperature was 240°C with the interface set at 230°C and the ion source at 200°C. Helium was used as carrier gas with constant flow rate of 1 mL/min. The column temperature program started with 40°C and was ramped to 150°C at a rate of 7 °C/min and then to 230°C at a rate of 9 °C/min, and finally held at 230°C for 9 minutes. The total run time was 40 minutes. SCFA were identified based on the retention time of standard compounds and with the assistance of the NIST 08 mass spectral library. Full scan mass spectra were recorded in the 25-150 m/z range (0.5 s/scan). Quantification was done by integration of the extracted ion chromatogram peaks for the following ion species: m/z 45 for acetate eluted at 7.8 minutes, m/z 74 for propionate eluted at 9.6 minutes, and m/z 60 for butyrate eluted at 11.5 minutes. GCMS solution software was used for data processing.

#### Plasmids

pLifeAct_mScarlet-i_N1 (Addgene plasmid # 85056 ; http://n2t.net/addgene:85056; RRID:Addgene_85056) and pmScarlet-H_C1 (Addgene plasmid # 85043; http://n2t.net/addgene:85043 ; RRID:Addgene_85043) was a gift from Dorus Gadella.^65^ CTTN_KR plasmid was synthesized and cloned into pcDNA3.1plus vector by GeneArt™ Thermo Fisher with previously reported mutations.^36^ pGFP Cortactin was a gift from Kenneth Yamada (Addgene plasmid # 50728 ; http://n2t.net/addgene:50728 ; RRID:Addgene_50728).^66^

### Quantification and statistical analysis

Data are expressed as mean±SD unless otherwise indicated in the figure legend. Analysis was performed using a two-sided Student’s t test, single comparison or analysis of variance test for multiple comparisons (one-way or two-way ANOVA followed by Tu’ey's or Bonferr’ni's multiple comparisons test, respectively). All experiments were conducted at least two times, unless otherwise indicated in the figure legends. n-numbers denote number of individual animals or cells isolated from individual animals. P-values of 0.05 were considered significant and are shown as p < 0.05 (∗), p < 0.01 (∗∗), p < 0.001 (∗∗∗), p < 0.0001 (∗∗∗∗). Graph generation and statistical analyses were performed using Prism version 8 software (GraphPad).

Statistical difference was determined by Student’s two-tailed t-test (Figures 1, 3A, 3B, 3D, 3G, 3M, 3N, 4, and S4F), by one-way ANOVA (Figures 2A, 2B, 3C, 3E, 3I, and 3J), area under the curve analysis (Figure 3K) and two-way ANOVA (Figure S4E).

Statistical analyses of panel Figure 2C were carried out using a one-dimensional linear mixed model that incorporates fixed effect parameters and random effects to analyze differences between cell subsets and replicate variances, respectively.

## Data Availability

• Proteomics data are available via ProteomeXchange with identifier PXD037723. Gene expression data is available on Gene Expression Omnibus with dataset identifier GSE216415. Original Western Blot images are included in Supplemental Information. Microscopy data reported in this paper will be shared by the lead contact upon request. • This paper does not report original code. • The data that support the findings of this study are available from the corresponding author upon reasonable request by email to mario.zaiss@uk-erlangen.de.
