# Supplementary material for: Alcohol-sourced acetate impairs T cell function by promoting cortactin acetylation
Source: iScience. 2023 Jun 28;26(7):107230. doi: 10.1016/j.isci.2023.107230 (PMC10362326; doi:10.1016/j.isci.2023.107230)
Supplement: Document S1. Figures S1–S6 [file mmc1.pdf]

iScience, Volume *26*

Supplemental information

Alcohol-sourced acetate impairs T cell function by promoting cortactin acetylation

Vugar Azizov, Michel Hübner, Michael Frech, Jörg Hofmann, Marketa Kubankova, Dennis Lapuente, Matthias Tenbusch, Jochen Guck, Georg Schett, and Mario M. Zaiss

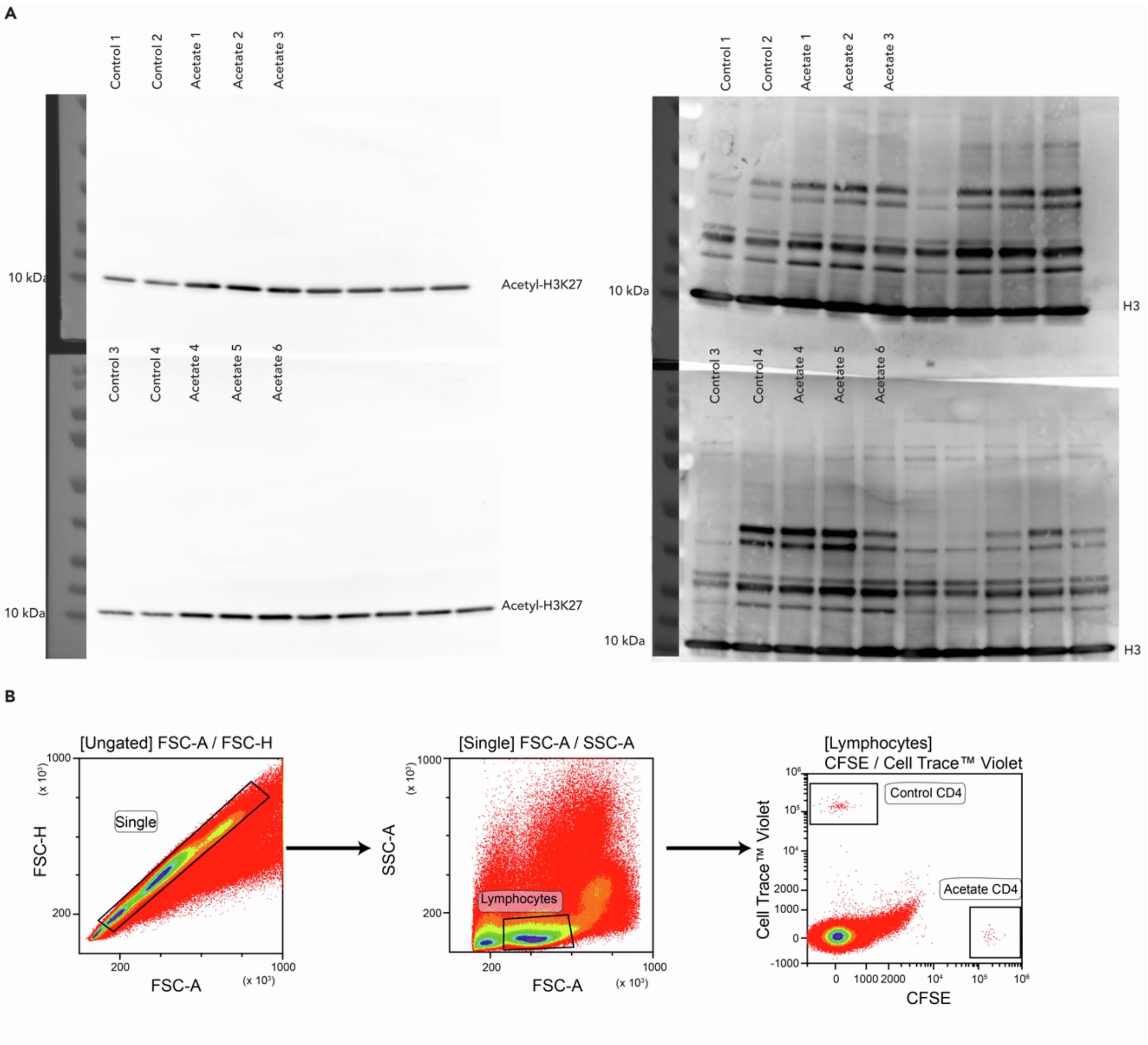

Figure S1. Exposure to acetate increases intracellular protein acetylation levels and hinders migration of T cells in adoptive transfer experiment, related to Figure 1. (**A**) Western Blot analysis of H3K27-acetyl levels, normalized to H3 levels in primary mouse CD4^{+} T cells. (**B**) Flow cytometry gating strategy for adoptive transfer of control (loaded with Cell Trace Violet) or acetate treated (loaded with CFSE) primary CD4^{+} T cell in C57BL/6 naïve mice.

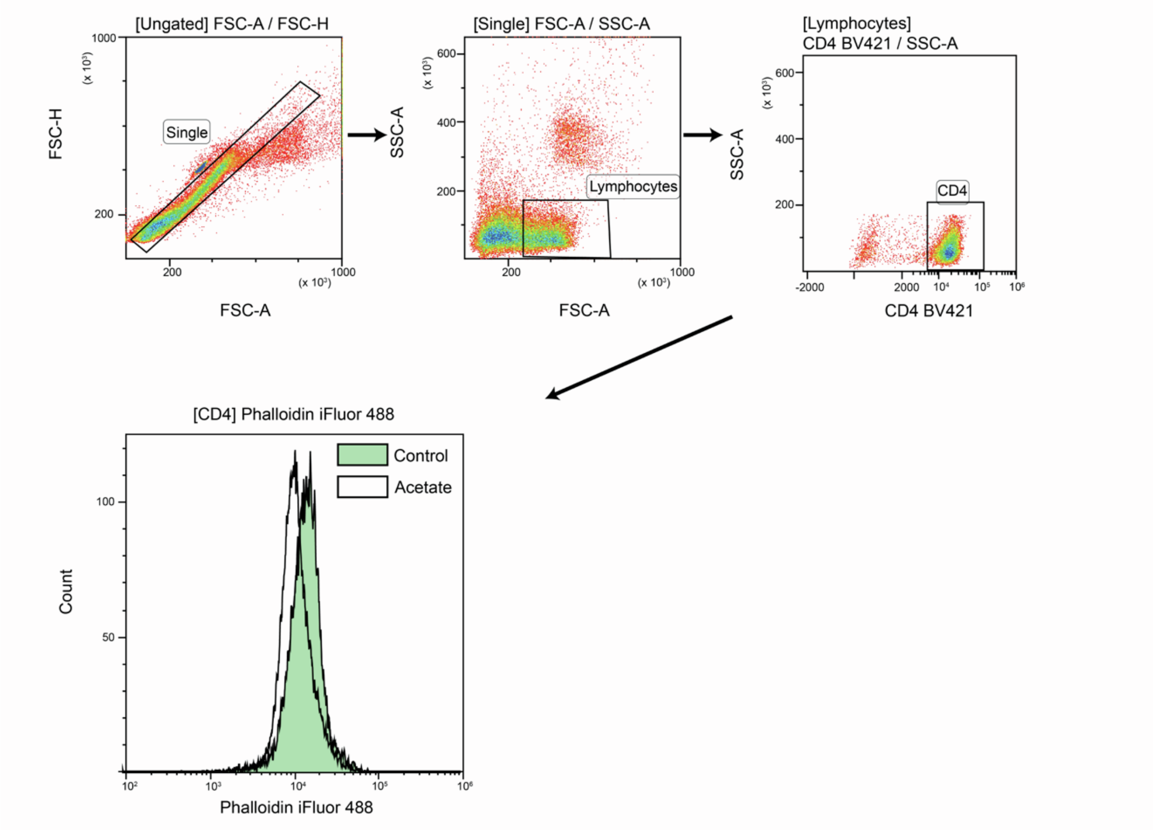

Figure S2. Analysis of F-actin levels by flow cytometry, related to Figure 2. Primary CD4+ T cells were isolated from the spleens of mice and upon in vitro experimentations were probed by phalloidin iFluor 488 and analyzed by flow cytometry. Median Fluorescence Intensity (MFI) were used throughout the manuscript.

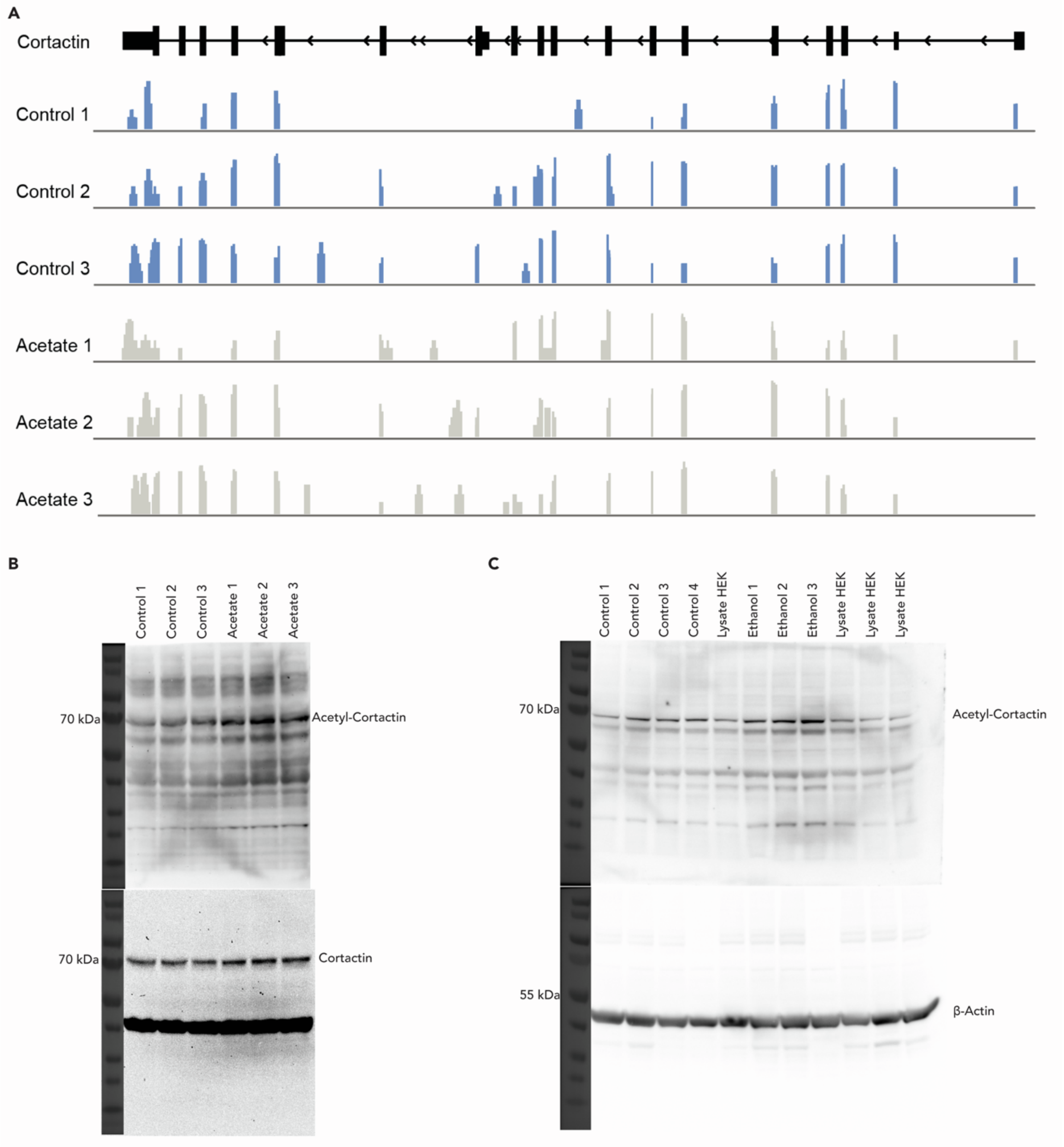

Figure S3. Cortactin is expressed in primary mouse CD4+ T cells, related to Figure 3. (**A**) Read peaks from RNA-sequencing in the cortactin gene in control (blue) and acetate-treated (grey) T cells are shown. (**B**) Western blot analysis of cortactin and acetylated cortactin in Jurkat T cells upon treatment with 5 mM acetate in vitro. (**C**) Western blot analysis of acetyl-cortactin in mice fed with alcohol (control n = 4, and alcohol n = 3).

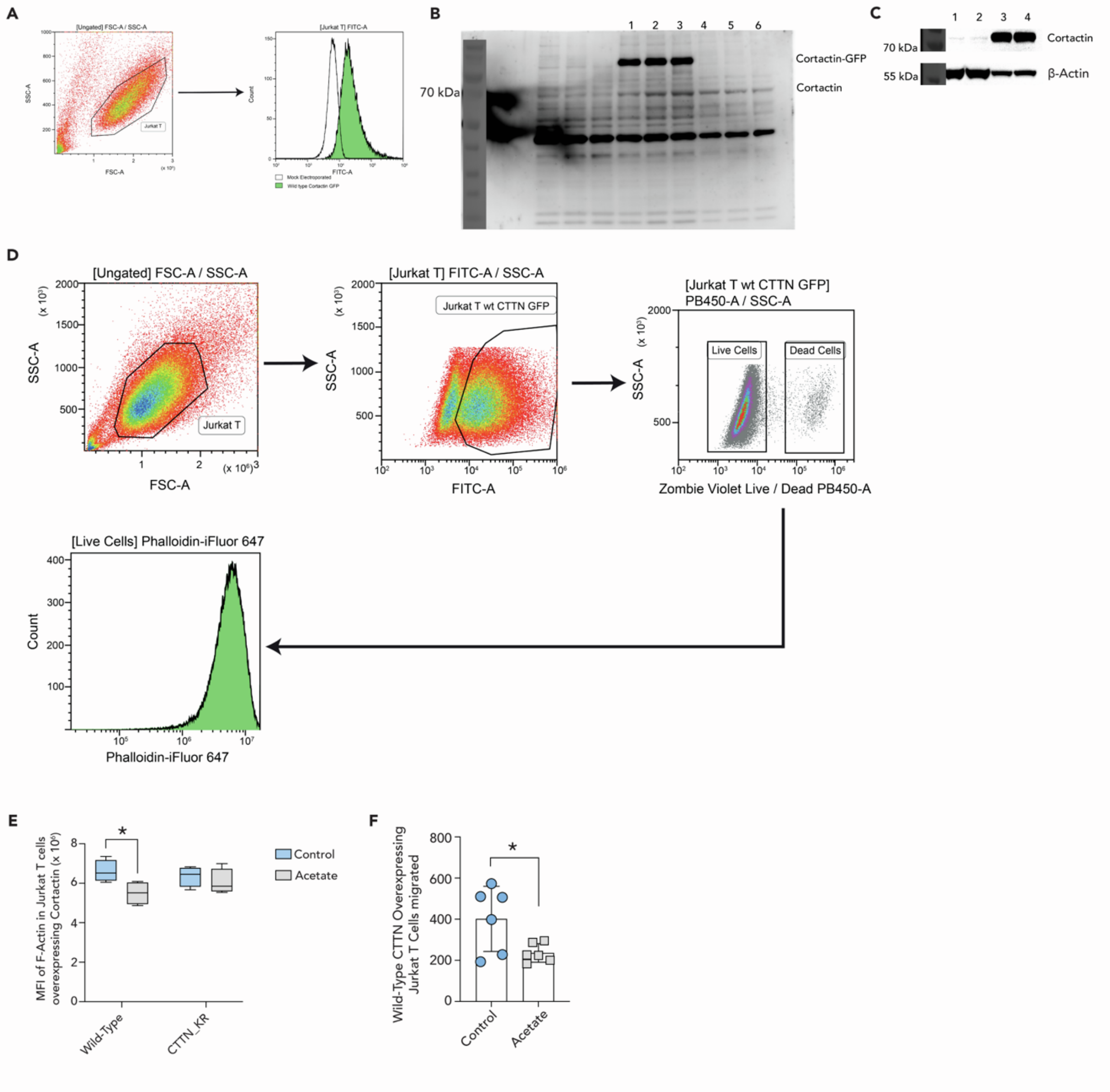

Figure S4. The effect of overexpression of wild type cortactin-GFP or CTTN_KR, related to Figure 3. (**A**) Analysis of electroporation of Jurkat T cells with wild type cortactin GFP overexpression plasmid (green) vs mock (blank line). (**B**) Western blot analysis of wild type cortactin GFP expression (lanes 1,2,3) and control (lanes 4,5,6) for cortactin. (**C**) Western blot analysis of CTTN_KR overexpression in Jurkat T cells. (**D**) Flow cytometry analysis of F-actin levels in Jurkat T cells (additional step of selecting GFP+ cells for wild type cortactin overexpressing cells) by phalloidin iFluor 647 probe. (**E**) F-actin MFI values determined by flow cytometry for Jurkat T cells overexpressing wild type cortactin GFP (Wild-Type) and lysine to arginine mutant CTTN_KR (CTTN_KR) upon treatment by 5 mM acetate. (**F**) Transwell migration assay of Jurkat T cell overexpressing wild type cortactin GFP upon treatment by 5 mM acetate. Representative data are shown from two independent experiments (E, F, G) and expressed as mean ± SD. Statistical difference was determined by Student's two-tailed t-test (F) or two-way ANOVA (E). $*p < 0.05$; $**p < 0.01$; $***p < 0.001$; $****p < 0.0001$.

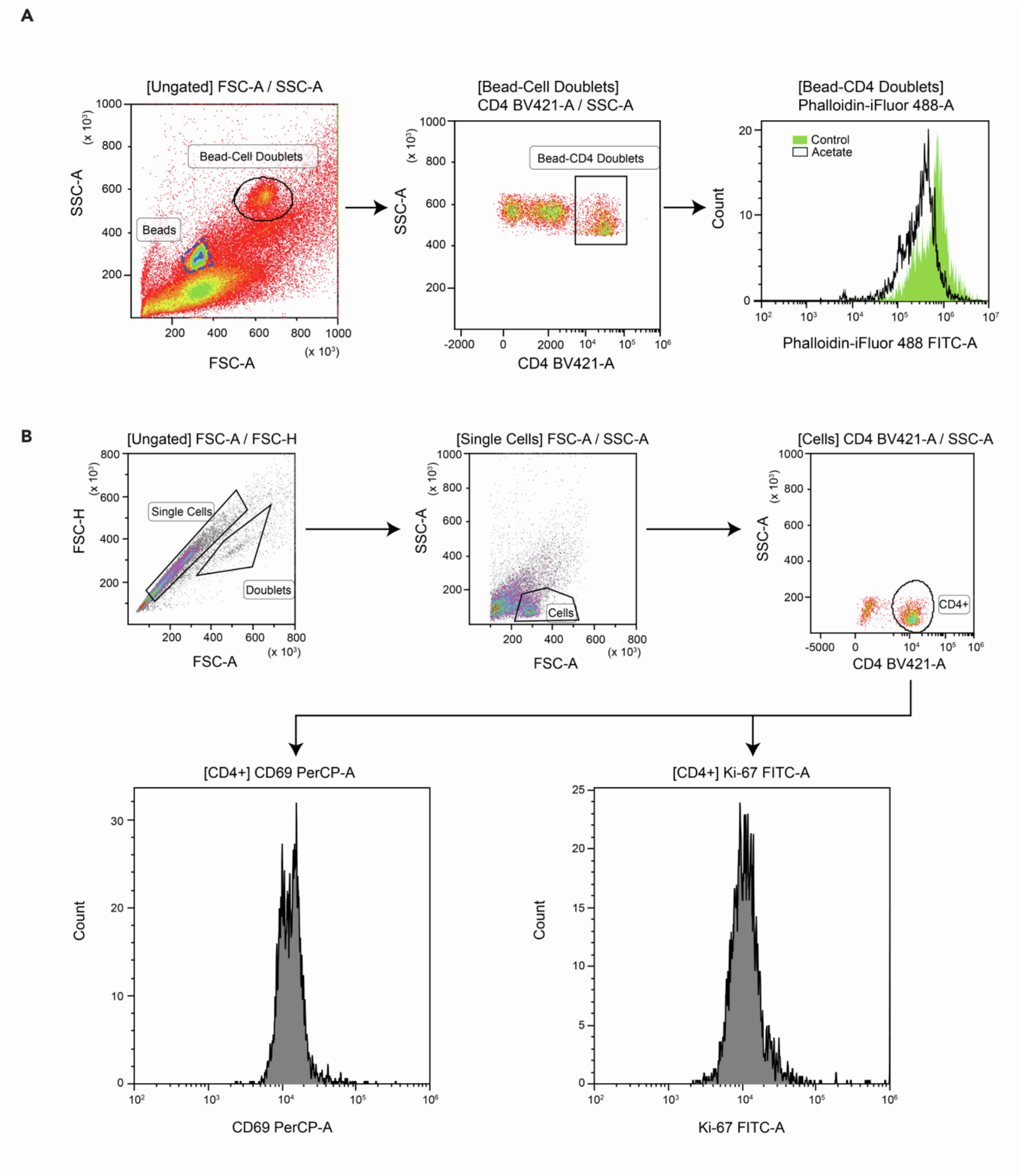

Figure S5. Flow cytometry analysis of T-Bead conjugates and T cell activation and proliferation markers, related to Figure 4. (**A**) Flow cytometry analysis F-actin levels of T-Bead conjugates where T cells were stained with anti-CD4 BV421 and were activated by beads coated with anti-CD3, anti-CD28 activating antibodies. F-actin levels were determined by phalloidin iFluor 488 MFI. (**B**) Flow cytometry analysis of T cells for CD69 and Ki67 levels activated in in vitro co-culture experiments.

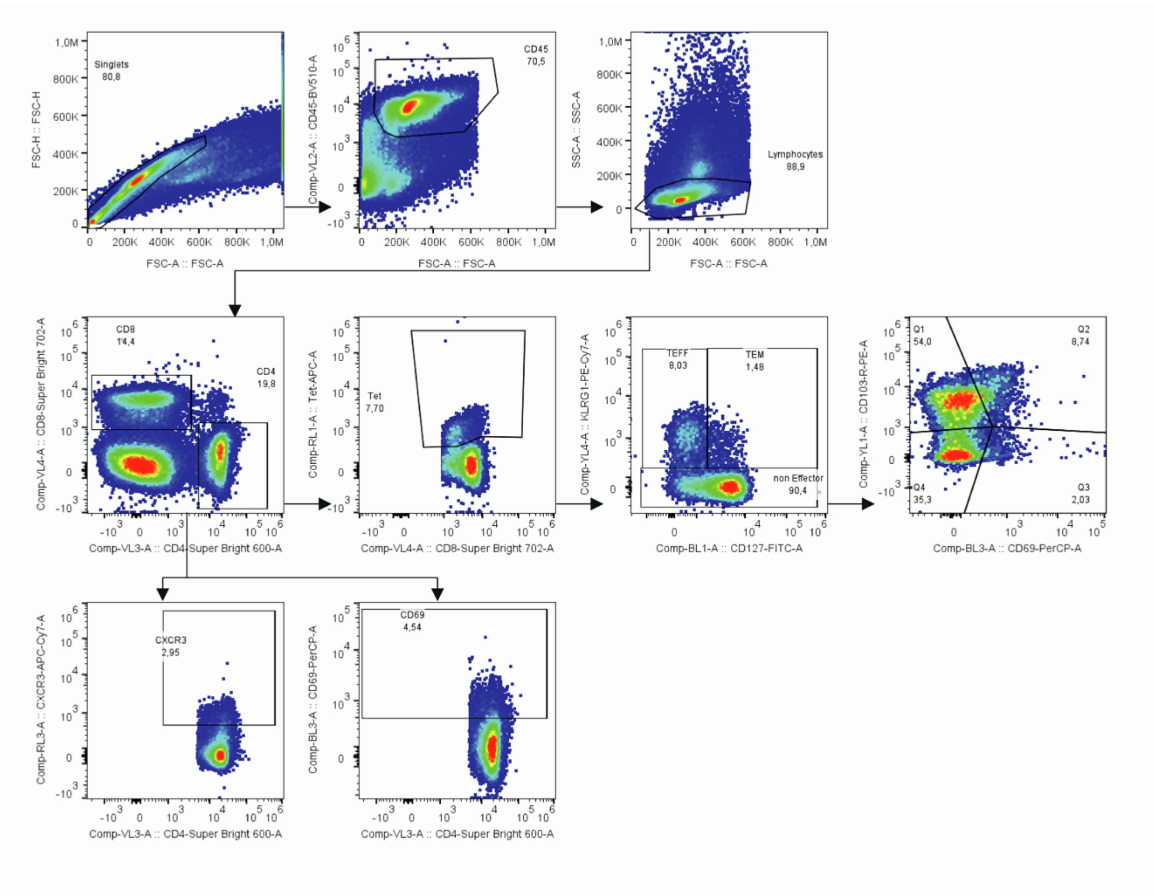

Figure S6. Flow cytometry analysis of T cells in the lungs of influenza infected alcohol consuming mice. Related to Figure 4. Gating strategy for subsets of CD8^{+} and CD4^{+} T cells isolated from the lungs of control mice (n = 6) and alcohol fed mice (n = 5).
